# A systematic review and meta-analysis of the protective effects of metformin in experimental myocardial infarction

**DOI:** 10.1371/journal.pone.0183664

**Published:** 2017-08-23

**Authors:** Nienke A. Hesen, Niels P. Riksen, Bart Aalders, Merel Ritskes-Hoitinga, Saloua El Messaoudi, Kimberley E. Wever

**Affiliations:** 1 SYstematic Review Centre for Laboratory animal Experimentation (SYRCLE), Department for Health Evidence, Nijmegen Institute for Health Sciences, Radboud University Medical Center, Nijmegen, The Netherlands; 2 Department of Internal Medicine, Radboud University Medical Center, Nijmegen, The Netherlands; 3 Department of Cardiology, Radboud University Medical Center, Nijmegen, The Netherlands; University of Colorado Denver, UNITED STATES

## Abstract

Metformin improves cardiovascular prognosis in patients with diabetes mellitus, compared to alternative glucose-lowering drugs, despite similar glycemic control. Direct cardiovascular protective properties have therefore been proposed, and studied in preclinical models of myocardial infarction. We now aim to critically assess the quality and outcome of these studies. We present a systematic review, quality assessment and meta-analysis of the effect of metformin in animal studies of experimental myocardial infarction. Through a comprehensive search in Pubmed and EMBASE, we identified 27 studies, 11 reporting on *ex vivo* experiments and 18 reporting on *in vivo* experiments. The primary endpoint infarct size as percentage of area at risk was significantly reduced by metformin *in vivo* (MD -18.11[-24.09,-12.14]) and *ex vivo* (MD -18.70[-25.39, -12.02]). Metformin improved the secondary endpoints left ventricular ejection fraction (LVEF) and left ventricular end systolic diameter. A borderline significant effect on mortality was observed, and there was no overall effect on cardiac hypertrophy. Subgroup analyses could be performed for comorbidity and timing of treatment (infarct size and mortality) and species and duration of ischemia (LVEF), but none of these variables accounted for significant amounts of heterogeneity. Reporting of possible sources of bias was extremely poor, including randomization (reported in 63%), blinding (33%), and sample size calculation (0%). As a result, risk of bias (assessed using SYRCLE’s risk of bias tool) was unclear in the vast majority of studies. We conclude that metformin limits infarct-size and improves cardiac function in animal models of myocardial infarction, but our confidence in the evidence is lowered by the unclear risk of bias and residual unexplained heterogeneity. We recommend an adequately powered, high quality confirmatory animal study to precede a randomized controlled trial of acute administration of metformin in patients undergoing reperfusion for acute myocardial infarction.

## Introduction

Already introduced in 1957 as antihyperglycemic drug, metformin is still the cornerstone of antidiabetic treatment in patients with type 2 diabetes mellitus. The United Kingdom Prospective Diabetes Study[1] reported that cardiovascular mortality and morbidity was lower in patients treated with metformin compared to alternative glucose-lowering drugs, despite similar glycemic control. It has been hypothesized ever since that metformin has direct cardioprotective properties independent from its glucose-lowering effect, and a wealth of preclinical studies have investigated such actions (reviewed in [2, 3]).

Indeed, in animal models of myocardial infarction, the administration of metformin potently limits ischemia-reperfusion injury and reduces infarct size, also in animals without diabetes (reviewed in [2]). In addition, long-term administration of metformin following coronary occlusion improves myocardial function in various animal studies. Unfortunately, translation of these promising preclinical findings to the clinical situation has been disappointing. In recent randomized controlled trials in patients without diabetes, treatment with metformin did not limit myocardial ischemia-reperfusion injury during coronary artery bypass grafting [4], nor cardiac remodelling in patients following myocardial infarction [5]. These disappointing findings urge a critical reappraisal of the animal studies on which the hypothesis that metformin has direct cardioprotective properties are based. In general, failed translation of preclinical results can be due to limited internal validity of the animal studies, limited external validity, or publication bias [6].

In systematic reviews and meta-analyses of animal studies on ischemic preconditioning of the kidney and the heart, we recently reported major flaws in the reporting of the experimental design and data analysis in the majority of the animal studies [7, 8]. In the current study, we aim to critically appraise the effect of metformin on myocardial ischemia-reperfusion injury in animal models of myocardial ischemia, and the quality of the reported data. Therefore, we performed a systematic review and meta-analysis of all animal studies investigating the effect of metformin administration on myocardial ischemia-reperfusion injury.

## Materials and methods

The review methodology was specified in advance and documented using SYRCLE’s systematic review protocol for animal intervention studies (see [9] and S1 File, registered on www.syrcle.nl on October 20^th^ 2015). The review question was: what is the effect of metformin treatment on cardiac damage and cardiac function in experimental myocardial infarction?

### Amendments to the review protocol

Due to software restrictions, heterogeneity was assessed using I^2^ and τ^2^, rather than R^2^. Because of inherent differences in (patho)physiology between *ex vivo* (isolated, perfused hearts) and *in vivo* models, we decided not to pool these models in any of the analyses. After close examination of the study characteristics, we hypothesized that the duration of myocardial ischemia (permanent *versus* temporary) was likely to explain part of the heterogeneity in our meta-analysis. We therefore added this variable to the subgroup analysis post-hoc.

### Study identification

Preclinical studies on the effects of metformin in experimental myocardial infarction were identified by comprehensive searches in two online databases (PubMed and EMBASE), using the search components “metformin”, “heart”, “ischemia” and “animal[10, 11]” (full search strategy in S2 File). Searches were performed on May 12^th^ 2017. No limits were applied for language or date. The reference lists of included studies and relevant reviews were hand searched to identify additional relevant studies.

### Study selection

References were exported to Endnote for removal of duplicates and were subsequently screened for relevance against pre-defined inclusion and exclusion criteria, using EROS (Early Review Organising Software; http://www.eros-systematic-review.org/). Subsequently, the full-text manuscripts of eligible studies were reviewed for final inclusion. We included animal studies using myocardial ischemia models (*in vivo* or *ex vivo*) with or without reperfusion, comparing metformin-treated animals with untreated or vehicle treated controls, and reporting any outcome related to myocardial damage or cardiac function in. In both selection phases, references were evaluated by two independent reviewers (NH and BA or NH and KW).

### Extraction of study characteristics and outcome data

Study characteristics were extracted by one reviewer (NH) and checked for inconsistencies by a second reviewer (KW). For each study, we extracted data on the animal model used (species, strain, sex, comorbidities, type of myocardial ischemia model, duration of ischemia and duration of reperfusion), the metformin treatment (administration route, timing and dose), as well as bibliographic details (1^st^ author, year, title, journal and language).

For each outcome measure, we identified all individual comparisons in which a group of animals receiving metformin was compared to a control group. Our primary outcome was the infarct size measured as a percentage of the area at risk (IS/AAR%). Secondary outcomes were circulating troponin levels, the left ventricular ejection fraction (LVEF%), the left ventricular end-systolic diameter (LVESD), cardiac hypertrophy and mortality. For all outcomes except mortality, data were extracted as mean, the standard deviation (SD) and number of animals per group (N). Mortality data were extracted as incidences out of total number of animals.

When data were presented graphically only, they were extracted using a digital screen ruler (ImageJ version 1.46r, National Institutes of Health, USA). Because of incomplete outcome data, we contacted the corresponding and lead author(s) of six studies via e-mail, with a request for additional information. We received a response from five authors, all of which were able to supply the missing data. If no response was received, the study/comparison was excluded from the analysis. In case the number of animals was unclear and could not be retrieved, a conservative estimate was made. Multiple treatment regimens from one study were extracted as separate comparisons. In such a case, the number of animals in the control group was adjusted for multiple comparisons. Data was extracted by one reviewer (NH) and a second reviewer checked the data for inconsistencies (KW).

### Study quality and risk of bias assessment

Two reviewers (NH and BA) independently assessed the risk of bias and study quality of each included study. In case of discrepancies, consensus was reached by discussion with a third reviewer (KW). Risk of bias was assessed using SYRCLE’s Risk of Bias tool [12]. The risk of bias due to selective outcome reporting (item #9) was not assessed, since none of the studies reported the use of a study protocol predefining primary and secondary outcomes. When assessing selection bias, groups within a study were considered similar at baseline if their sex, baseline blood pressure and heart rate did not significantly differ. In addition, if the IS/AAR% was reported, we assessed whether the AAR was similar between the groups. To assess whether studies were free of other risks of bias, addition of animals to groups during the experiment and a possible conflict of interest were taken into account. We also assessed reporting of the following study quality indicators: any randomization, any blinding, regulation of body temperature within 3°C variation, sample size calculation and a conflict of interest statement.

### Data synthesis

For outcomes reported by at least three studies, meta-analysis was performed using Review Manager (RevMan; version 5.3.5; Copenhagen: The Nordic Cochrane Centre, The Cochrane Collaboration, 2014). For IS/AAR% and LVEF%, the raw mean difference (MD) was calculated. For the circulating troponin, LVESD and cardiac hypertrophy, we aimed to calculate the normalised mean difference (NMD) if sham data were available for the majority of studies. However, since this was not the case, the standardised mean difference (SMD) was used. Mortality data were analyzed as odds ratios (OR). Data were pooled using a random effects model to account for expected between-study heterogeneity. To assess heterogeneity, the I^2^ and τ^2^ statistics were determined. To examine potential sources of heterogeneity, predefined subgroup analyses were performed when subgroups contained data from at least three studies. Differences between subgroups were tested using the standard function in RevMan, which tests the heterogeneity across subgroups against a χ^2^ distribution with 1−*S* degrees of freedom (where *S* is the number of subgroups with summary effect sizes). For each outcome measure, the significance level for subgroup analyses was adjusted for the number of analyses using the Bonferroni-Holm method [13]. Results are reported as a MD, SMD or OR with their 95% confidence interval [95%CI], unless stated otherwise.

The planned sensitivity analysis regarding the time point of outcome measurement was not performed, since none of the studies reported our selected outcomes at more than one time-point. Sensitivity analysis using the SMD instead of the NMD for continuous outcomes was omitted since none of the outcome measures could be analyzed as NMD. We performed sensitivity analysis on the robustness of our findings on mortality using the OR by re-analysing these data using the risk ratio (RR).

One study reported cardiac hypertrophy separately for the right ventricle, the left ventricle and the atrium. Because these outcomes were all measured in the same animals and are therefore not independent, we included data for the left ventricle/body weight ratio in the main analysis, and performed sensitivity analysis by substituting with respectively the right ventricle/body weight and atrium/body weight.

A descriptive summary is provided for outcome measures reported by fewer than three studies, which was the case for circulating troponin.

## Results

### Search and study selection

**Fig 1:**
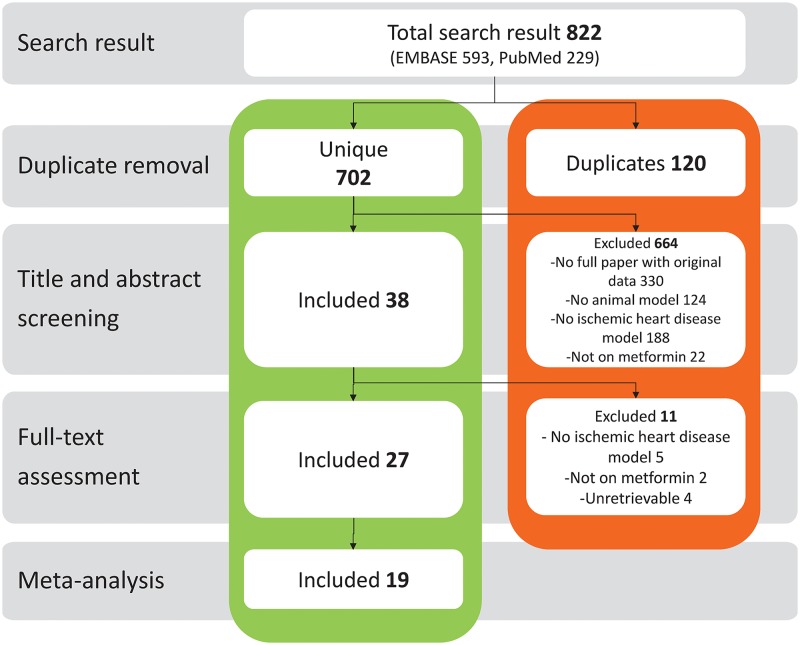
Flow chart of the study identification and selection process.

The comprehensive search identified 566 unique references, 535 of which were excluded after screening on title and abstract (see Fig 1). Another eleven references were excluded after assessing the full-text manuscript, resulting in 27 studies being included in the systematic review. Nineteen studies reported one or more of our predefined outcome measures and were included in the meta-analysis.

### Study characteristics

Characteristics of the 27 included studies are summarized in Table 1 (*ex vivo* models) and Table 2 (*in vivo* models). Fifteen studies (56%) were conducted in rats or isolated rat hearts, two (7%) in isolated rabbit hearts, seven (26%) in mice and three studies (26%) were performed in pigs. Fourteen studies (52%) used healthy animals only. Separate groups of diabetic animals were included in eight studies (30%), and in five studies (19%) animals were fed a high-fat diet as a model for insulin resistance or metabolic syndrome. Twenty-four studies (89%) were performed in male animals, and in the remaining three studies the sex of the animals was not reported. Eleven publications reported data from *ex vivo* models, and eighteen *in vivo* studies were identified, two of which were cardiac transplantation models. Myocardial ischemia was permanent in eleven of the *in vivo* models, and transient in seven (including the two cardiac transplantation models). All *ex vivo* models used transient ischemia, except for the two conducted in rabbits.

**Table 1:** Study characteristics of ex vivo models.

Article	Species (strain)	Co-morb	Sex	I	R	Admin	Metformin start	Metformin dose	Metformin duration	OMs included in MA
BarretoTorres2012 [14]	rat (SD)	None	m	30	30	perfusate	10min pre I	2 mM	70min	NR
Bhamra2008 [15]	rat (W)	None	m	35	120	perfusate	at onset R	25/ 50/ 75 umol/l	15min	IS/AAR
	rat (GK)	DM	m	35	120	perfusate	at onset R	50 umol/l	15min	IS/AAR
Kawabata2003a [16]	rabbit (JW)	None	m	45	none	*i*.*v*.	60min pre I	0.14 mg/kg	1d 1x/d	NR
Kawabata2003b [17]	rabbit (JW)	None	m	45	none	*i*.*v*.	60min pre I	0.14 mg/kg	1d 1x/d	NR
Kravchuk2011 [18]	rat (W)	None	m	30	120	*i*.*p*.	3d pre I	200 mg/kg	3d 1x/d	NR
	rat (W)	DM	m	30	120	*i*.*p*.	3d pre I	200 mg/kg	3d 1x/d	NR
Legtenberg2002 [19]	rat (W)	None	m	12	28	perfusate	10min pre I	50/ 500 umol/l	42min	NR
Paiva2009 [20]	rat (W)	None	m	35	120	perfusate	at onset R	50 uM	15min	IS/AAR
Paiva2010 [21]	rat (W)	None	m	35	120	perfusate	at onset R	50 uM	15min	IS/AAR
Sauve2010 [22]	mouse (C57BL/6J)	None	m	30	40	*i*.*p*.	1d pre I	125ug/kg	2d 1x/d	NR
Solskov2008 [23]	rat (W)	None	m	45	120	*p*.*o*.	24h pre I	250 mg/kg	1d 1x/d	IS/AAR
Whittington2013 [24]	rat (W)	None	m	35	60	*p*.*o*.	4wk pre I	300 mg/kg	4wk 1x/d	IS/AAR
	rat (GK)	DM	m	35	60	*p*.*o*.	4wk pre I	300 mg/kg	4wk 1x/d	IS/AAR

Co-morb = co-morbidity; I = duration of myocardial ischemia; R = duration of reperfusion; admin = administration route; OM = outcome measure; MA = meta-analysis; SD = Sprague-Dawley; W = wistar; GK = Goto Kakizaki; JW = Japanese white; DM = diabetes; m = male; NR = not reported; d = day(s); wk = week(s); i.v. = intra-veneously; i.p. intra-peritoneally; p.o. = per os; IS/AAR = infarct size/area at risk; I and R duration are given in minutes unless indicated otherwise

**Table 2:** Study characteristics of in vivo models.

Article	Species (strain)	Co-morb	Sex	I	R	Admin	Metformin start	Metformin dose	Metformin duration	OMs included in MA
Apaijai2014 [25]	rat (W)	None	m	30	120	*p*.*o*.	21d pre I	30 mg/kg	21d 1x/d	IS/AAR, LVEF, Mortality
	rat (W)	HFD	m	30	120	*p*.*o*.	21d pre I	30 mg/kg	21d 1x/d	IS/AAR, LVEF, mortality
Apaijai2016	rat (W)	None	m	8wk	none	*p*.*o*.	at onset I	30 mg/kg	8wk 1x/d	LVEF, hypertrophy
	rat (W)	HFD	m	8wk	none	*p*.*o*.	at onset I	30 mg/kg	8wk 1x/d	LVEF, hypertrophy
Calvert2008 [26]	mouse (C57BL6/J)	None	m	30	≥240	*i*.*p*./ *i*.*c*.	18h pre I / at onset R	125ug/kg	1d 1x/d	IS/AAR, LVEF, LVESD
	mouse (Lepr ^db^/J)	DM	m	30	≥240	*i*.*p*./ *i*.*c*.	18h pre I / at onset R	125ug/kg	1d 1x/d	IS/AAR
Cantin2002 [27]	rat (Lewis)	DM	m	Tx iso	30/ 60d	*p*.*o*.	6d post Tx	500 mg/kg	24/ 54d 2x/d	NR
	rat (Lewis)	DM	m	Tx allo	30/ 60d	*p*.*o*.	6d post Tx	500 mg/kg	24/ 54d 2x/d	NR
Chin2011 [28]	mouse (C57BL/6J)	None	NR	Tx allo		*i*.*p*. + *i*.*v*. + immersion	1h pre Tx	NR	1d 3x/d / 1d 3x/d + 8d 0.5x/d	NR
Elmadhun2013[29]	pig (O)	HFD	m	7wk*	none	*p*.*o*.	at onset I	500 mg	7wk 2x/d	Mortality
Elmadhun2014 [30]	pig (O)	HFD	m	7wk*	none	*p*.*o*.	at onset I	500 mg	7wk 2x/d	NR
Gundewar2009 [31]	mouse (C57BL/6J)	None	NR	4wk	none	*i*.*p*.	at onset I	125ug/kg	4wk 1x/d	Mortality, LVEF
	mouse (C57BL/6J)	None	NR	60	4wk	*i*.*c*./ *i*.*c*. + *i*.*p*.	at onset R	125ug/kg	1d 1x/d / 4wk 1x/d	IS/AAR, LVEF, LVESD, Hypertrophy
Inthachai2015 [32]	rat (W)	None	m	8wk	none	*p*.*o*.	3d post I	15mg/kg	8wk 2x/d	Hypertrophy
Lassaletta2012 [33]	pig (O)	HFD	NR	7wk*	none	*p*.*o*.	at onset I	500 mg	7wk 2x/d	NR
NoyanAshraf2009 [34]	mouse (C57BL/6J)	None	m	28d	none	*p*.*o*.	7d pre I	6.76 g/kg of chow	7d ad libitum	LVESD, Hypertrophy
Oidor-Chan2016	rat (W)	None	m	30	120	*p*.*o*.	14d pre I	300 mg/kg	14d 1x/d	IS/AAR
	rat (W)	DM	m	30	120	*p*.*o*.	14d pre I	300 mg/kg	14d 1x/d	IS/AAR
	mouse (C57BL/6J)	DM +HFD	m	28d	none	*p*.*o*.	7d pre I	6.76 g/kg of chow	7d ad libitum	mortality
Paiva2009 [20]	rat (SD)	None	m	30	120	*i*.*v*.	5min pre R	5mg/kg	1d 1x/d	IS/AAR
Sauve2010 [22]	mouse (C57BL/6J)	None	m	4/5d	none	*p*.*o*.	7d pre I	450 mg/kg	1wk 1x/d	LVESD
	mouse (C57BL/6J)	DM +HFD	m	4wk	none	*p*.*o*.	8wk pre I	450 mg/kg	12wk 1x/d	hypertrophy, mortality
Sun2017	mouse (C57BL/6)	None	m	4wk	none	*p*.*o*.	3d post I	200 mg/kg	4wk 1x/d	LVEF, LVESD
Wang2011 [35]	rat (W)	None	m	8wk	none	*p*.*o*.	4wk post I	100 mg/kg	4wk 1x/d	Mortality, LVEF, LVESD
Yin2011 [36]	rat (SD)	None	m	12wk	none	*p*.*o*.	2d pre I	250 mg/kg	12wk 1x/d	Hypertrophy, LVEF
Yue2016	mouse (C57)	None	m	4wk	none	*i*.*p*.	6h pre I	125 mg/kg	7d 1x/d	LVEF

Co-morb = co-morbidity; I = duration of myocardial ischemia; R = duration of reperfusion; admin = administration route; OM = outcome measure; MA = meta-analysis; SD = Sprague-Dawley; W = wistar; O = Ossabaw mini; DM = diabetes; HFD = high fat diet; m = male; NR = not reported; d = day(s); wk = week(s); i.v. = intra-venously; i.p. intra-peritoneally; p.o. = per os; i.c. = intra-cardiac; Tx = cardiac transplantation; iso = isogenic; allo = allogenic; IS/AAR = infarct size/area at risk; LVESD = left ventricle end systolic diameter; LVEF = left ventricular ejection fraction; I and R duration are given in minutes unless indicated otherwise;

*gradual ischemia by ameroid constrictor

A variety of treatment regimes was used. Metformin treatment was initiated before the onset of ischemia (fifteen studies), at the onset of ischemia (four studies), during permanent ischemia (four studies), at the onset of reperfusion (five studies) or later in the reperfusion phase (one study). The duration of treatment varied between 15 minutes of perfusion with metformin present in the perfusate, to a single bolus given just before the experiment, to daily dosages given for twelve weeks. Oral dosages between 15 and 500mg/kg were most common, but intra-peritoneal, intra-cardiac and intra-venous administration was also used.

Of our predefined outcome measures, the IS/AAR was reported in six *ex vivo* studies and five *in vivo* studies, all of which used temporary occlusion models. The LVEF, LVESD, cardiac hypertrophy and mortality were reported in respectively eight, six, six and seven *in vivo* studies, and circulating troponin in one *in vivo* study. The latter outcome measures were not reported in any of the *ex vivo* studies.

### Study quality and risk of bias assessment

**Fig 2:**
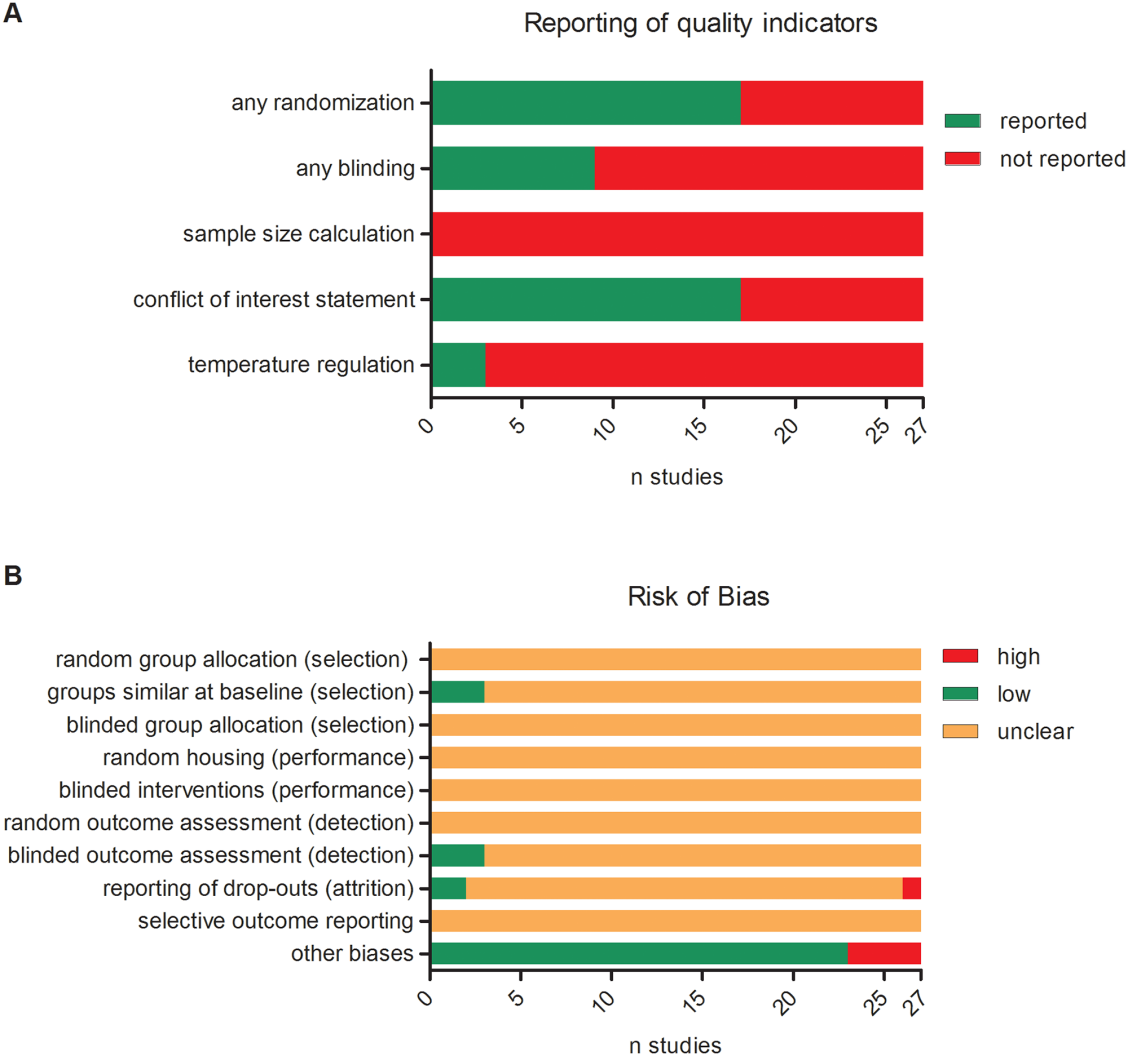
Risk of bias and quality assessment. Poor reporting of key study quality indicators (**A**) resulted in an unclear risk of bias for most types of bias (**B**).

The results of the study quality and risk of bias assessment are shown in Fig 2 (overall scores) and Table 3 (individual scores). Randomisation was mentioned in seventeen studies (63%; Fig 2A), but details regarding the randomisation procedure were not reported. As a result, the risk of bias due to random allocation, random housing and random assessment of outcome was unclear (Fig 2B). Nine studies (33%) mentioned that any blinding was applied during the experiment (Fig 2A), but this was always related to the outcome assessment (seven studies blinded some and two blinded all of the outcome measures). Thus, the risk of bias due to concealment of group allocation and blinding of personnel during the experiment was assessed as unclear (Fig 2B). Three studies reported sufficient detail to assess that the groups were similar at baseline, the other studies did not report all of the baseline characteristics we assessed (Fig 2B). Two studies reported sufficient detail to assess that the risk of attrition bias was low. In one study, the risk was assessed as high due to discrepancies between the group sizes mentioned in the results and those in the materials and methods. In all other studies, the number of animals per group was either mentioned only once, given as a range, or not mentioned at all, resulting in an unclear risk of attrition bias. The risk of selective outcome reporting was unclear in all studies, since none of them reported whether the primary and/or secondary outcomes were pre-specified in a protocol, and no study protocols were included with the publications. In four studies, the risk of other biases was assessed as high: two because of potential conflicts of interest, and two because drop-outs appeared to have been replaced with new animals.

**Table 3:** Individual risk of bias and quality scores.

	Risk of bias										Reporting				
Article ID	Random group allocation (selection)	Groups similar at baseline (selection)	Blinded group allocation (selection)	Random housing (performance)	Blinded interventions (performance)	Random outcome assessment (detection)	Blinded outcome assessment (detection)	Reporting of dropouts (attrition)	Selective outcome reporting	Other biases	Experiment randomised?	Experiment blinded?	Power analysis or sample size calculation?	Conflict of interest statement?	Body temperature maintained (3°C range)?
Apaijai2014[25]	?	?	?	?	?	?	?	?	?	L	N	N	N	Y	N
Apaijai2016	?	?	?	?	?	?	?	H	?	H	Y	N	N	Y	N
BarretoTorres2012[14]	?	?	?	?	?	?	?	?	?	L	Y	N	N	Y	N
Bhamra2008[15]	?	?	?	?	?	?	?	?	?	L	Y	N	N	N	Y
Calvert2008[26]	?	?	?	?	?	?	?	?	?	L	N	N	N	N	N
Cantin2002[27]	?	?	?	?	?	?	?	?	?	L	N	N	N	N	N
Chin2011[28]	?	?	?	?	?	?	?	?	?	L	N	N	N	N	N
Elmadhun2013[29]	?	?	?	?	?	?	?	L	?	H	N	Y	N	Y	N
Elmadhun2014[30]	?	?	?	?	?	?	?	?	?	L	Y	Y	N	Y	N
Gundewar2009[31]	?	?	?	?	?	?	?	?	?	L	Y	N	N	Y	N
Inthachai2015[32]	?	?	?	?	?	?	?	?	?	L	Y	N	N	Y	N
Kawabata2003a[16]	?	?	?	?	?	?	?	?	?	L	N	N	N	N	N
Kawabata2003b[17]	?	?	?	?	?	?	?	L	?	L	N	N	N	N	N
Kravchuk2011[18]	?	?	?	?	?	?	?	?	?	L	N	N	N	N	N
Lassaletta2012[33]	?	?	?	?	?	?	?	?	?	L	N	Y	N	Y	N
Legtenberg2002[19]	?	?	?	?	?	?	?	?	?	L	N	N	N	N	Y
NoyanAshraf2009[34]	?	?	?	?	?	?	?	?	?	H	Y	N	N	Y	N
Oidor-Chan2016	?	?	?	?	?	?	?	?	?	L	Y	N	N	Y	N
Paiva2009[20]	?	L	?	?	?	?	?	?	?	L	Y	N	N	Y	N
Paiva2010[21]	?	L	?	?	?	?	?	?	?	L	Y	N	N	Y	Y
Sauve2010[22]	?	?	?	?	?	?	?	?	?	H	Y	Y	N	Y	N
Solskov2008[23]	?	L	?	?	?	?	L	?	?	L	Y	Y	N	N	N
Sun2017	?	?	?	?	?	?	?	?	?	L	Y	N	N	Y	N
Wang2011[35]	?	?	?	?	?	?	L	?	?	L	Y	Y	N	N	N
Whittington2013[24]	?	?	?	?	?	?	?	?	?	L	Y	Y	N	Y	N
Yin2011[36]	?	?	?	?	?	?	?	?	?	L	Y	Y	N	Y	N
Yue2016	?	?	?	?	?	?	L	?	?	L	Y	Y	N	Y	N

? = unclear risk of bias; L = low risk of bias; H = high risk of bias; Y = yes; N = no

None of the studies reported a power analysis or sample size calculation justifying the number of animals per group (Fig 2A). Seventeen studies (63%) reported a conflict of interest statement, two of which described a potential conflict of interest. Three studies (11%) mentioned that body temperature during surgery was maintained within a 3°C range.

### Meta-analysis

#### Primary outcome: Infarct size

**Fig 3:**
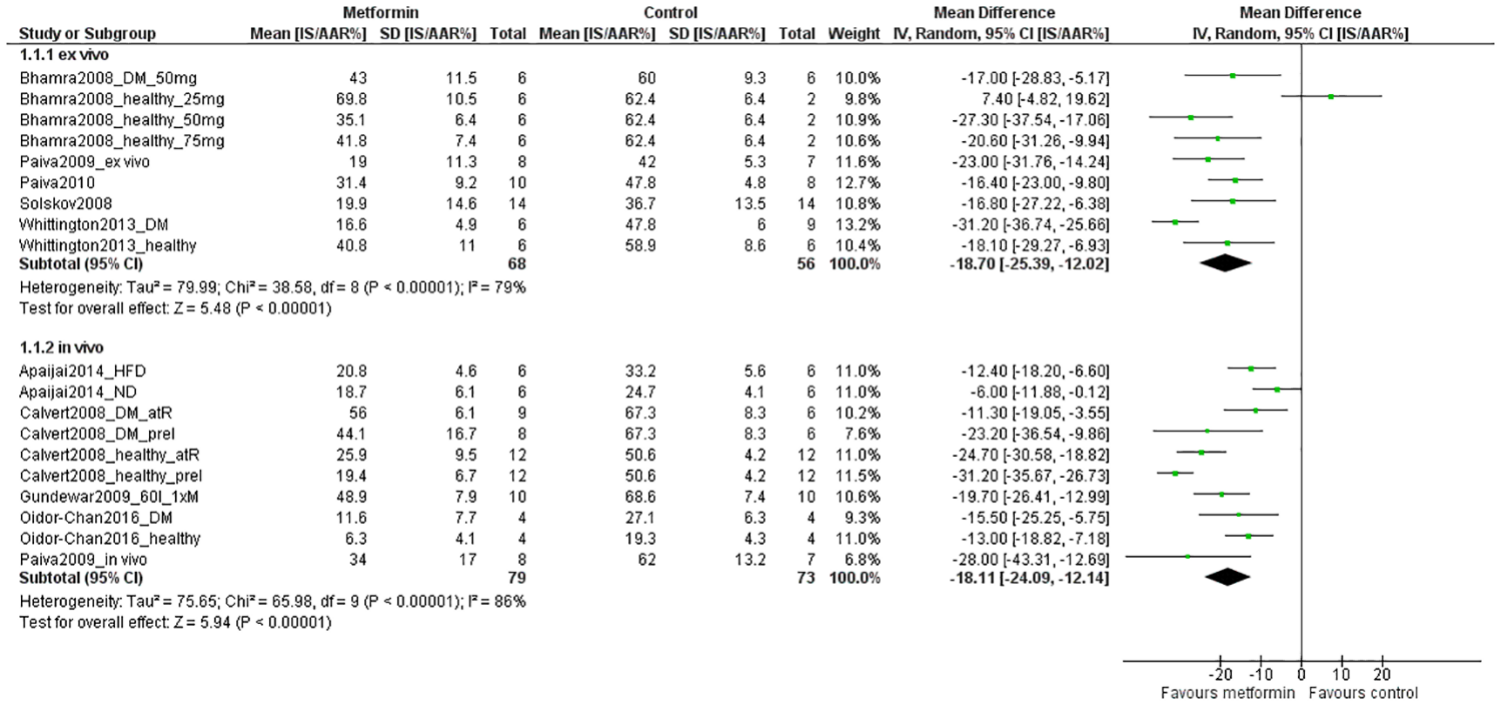
Forest plot of the effect of metformin on myocardial infarct size. Infarct size data expressed as a percentage of the area at risk (IS/AAR%) in metformin-treated groups and control groups were extracted from primary studies and expressed as mean differences (green squares). The pooled effect estimates (diamonds) show a significant reduction of infarct size in metformin-treated animals, in both *ex vivo* and *in vivo* models (pooled separately). SD = standard deviation, Total = sample size, CI = confidence interval, IV = inverse variance, HFD = high fat diet, ND = normal diet, DM = diabetes mellitus, permI = permanent ischemia, 60I = 60 minutes of ischemia, 1xM = single dose of metformin, 28xM = 28 doses of metformin in total, atR = treatment at reperfusion, preI = treatment pre-ischemia.

Data on the infarct size reported as IS/AAR% was analyzed separately for *ex vivo* and *in vivo* models (Fig 3). We could include data of nine comparisons from five studies and thirteen comparisons from seven studies for respectively *ex vivo* and *in vivo* models. As such, data from 68 metformin-treated isolated hearts and 56 control hearts were included in the *ex vivo* analysis, and 107 metformin-treated animals and 95 controls in the *in vivo* analysis. A significant reduction in IS/AAR% in metformin-treated groups was observed in both *ex vivo* models (MD -18.70[-25.39, -12.02]; P<0.00001) and *in vivo* models (MD -18.11[-24.09,-12.14]; P<0.0001). Between-study heterogeneity was high in both analyses: 79% and 86% in *ex vivo* and *in vivo* models, respectively.

Subgroup analysis for *in vivo* models showed no effect of comorbidity or timing of treatment on the effect of metformin on infarct size (figures A and B in S3 File). Subgroup analysis for the variables species, sex, frequency of treatment, duration of treatment, reporting of randomisation and reporting of blinding could not be preformed due to insufficient data (*i*.*e*. one or more of the subgroups contained <3 studies, and therefore no sensible comparison between subgroups could be made). Likewise, subgroup analyses could not be performed for *ex vivo* models.

#### Secondary outcomes: LVEF

**Fig 4:**
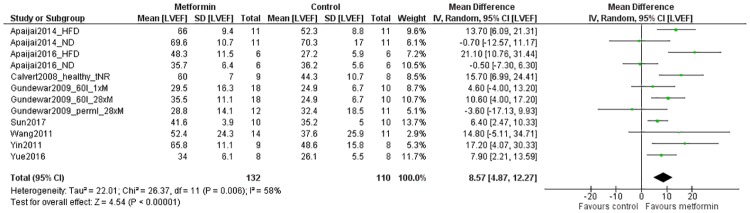
Forest plot of the effect of metformin on the left ventricular ejection fraction (LVEF) after myocardial infarction *in vivo*. Outcome data from metformin-treated groups and control groups were extracted from primary studies and expressed as mean differences (green squares). The bottom pooled effect estimate (diamond) shows a significant increase in the LVEF in metformin-treated animals. SD = standard deviation, Total = sample size, CI = confidence interval, IV = inverse variance, HFD = high fat diet, ND = normal diet, permI = permanent ischemia, 60I = 60 minutes of ischemia, 1xM = single dose of metformin, 28xM = 28 doses of metformin in total, tNR = time of treatment not reported.

The LVEF was reported in *in vivo* studies only. Data of twelve comparisons from eight studies could be included in the analysis, which were obtained from a total of 132 metformin-treated animals and 110 controls. Overall, metformin treatment increased the LVEF when compared to controls (MD 8.57 [4.87, 12.27]; P = 0.0001; Fig 4). There was moderate heterogeneity (58%; P = 0.006). Subgroup analyses could only be performed for species, and the duration of ischemia (post-hoc). These analyses indicated no difference in treatment efficacy between rat and mice, nor between permanent and temporary occlusion models (figures C and D in S3 File).

#### Secondary outcomes: LVESD

**Fig 5:**
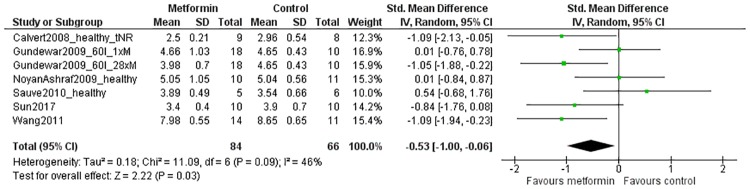
Forest plot of the effect of metformin on the left ventricular end-systolic diameter (LVESD) after myocardial infarction *in vivo*. Outcome data from metformin-treated groups and control groups were extracted from primary studies and expressed as standardized mean differences (green squares). The pooled effect estimate (diamond) indicates a reduction in LVESD in metformin-treated animals when compared to controls. SD = standard deviation, Total = sample size, CI = confidence interval, IV = inverse variance, 60I = 60 minutes of ischemia, 1xM = single dose of metformin, 28xM = 28 doses of metformin in total, tNR = time of treatment not reported.

The LVESD was reported in *in vivo* studies only. Data of seven comparisons from six studies could be included in the analysis, which were obtained from a total of 84 metformin-treated animals and 66 controls. The overall effect estimate indicated that metformin treatment reduced the LVESD (SMD -0.53[-1.00, -0.06]; P = 0.03; Fig 5). There was moderate to low heterogeneity (46%; P = 0.09). Subgroup analyses could not be performed due to insufficient data.

#### Secondary outcomes: Cardiac hypertrophy

**Fig 6:**
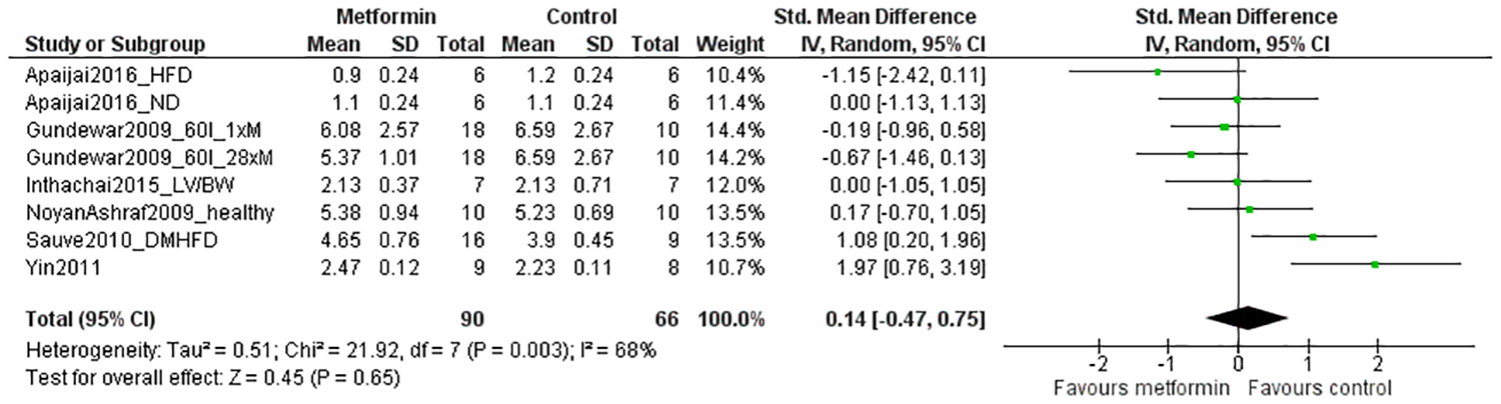
Forest plot of the effect of metformin on cardiac hypertrophy after myocardial infarction *in vivo*. Outcome data from metformin-treated groups and control groups were extracted from primary studies and expressed as standardized mean differences (green squares). The pooled effect estimate (diamond) indicates no difference in cardiac hypertrophy between metformin-treated animals and controls. SD = standard deviation, Total = sample size, CI = confidence interval, IV = inverse variance, 60I = 60 minutes of ischemia, 1xM = single dose of metformin, 28xM = 28 doses of metformin in total, LV/BW = left ventricle/body weight, DMHFD = diabetes mellitus and high-fat diet.

Cardiac hypertrophy was reported in *in vivo* studies only. Data of eight comparisons from six studies could be included in the analysis, which were obtained from a total of 90 metformin-treated animals and 66 controls. The overall effect estimate indicated no effect of metformin treatment on cardiac hypertrophy (SMD 0.14[-0.47, 0.75]; P = 0.65; Fig 6). There was substantial heterogeneity (68%; P = 0.003), but subgroup analyses could not be performed due to insufficient data.

#### Secondary outcomes: Mortality

**Fig 7:**
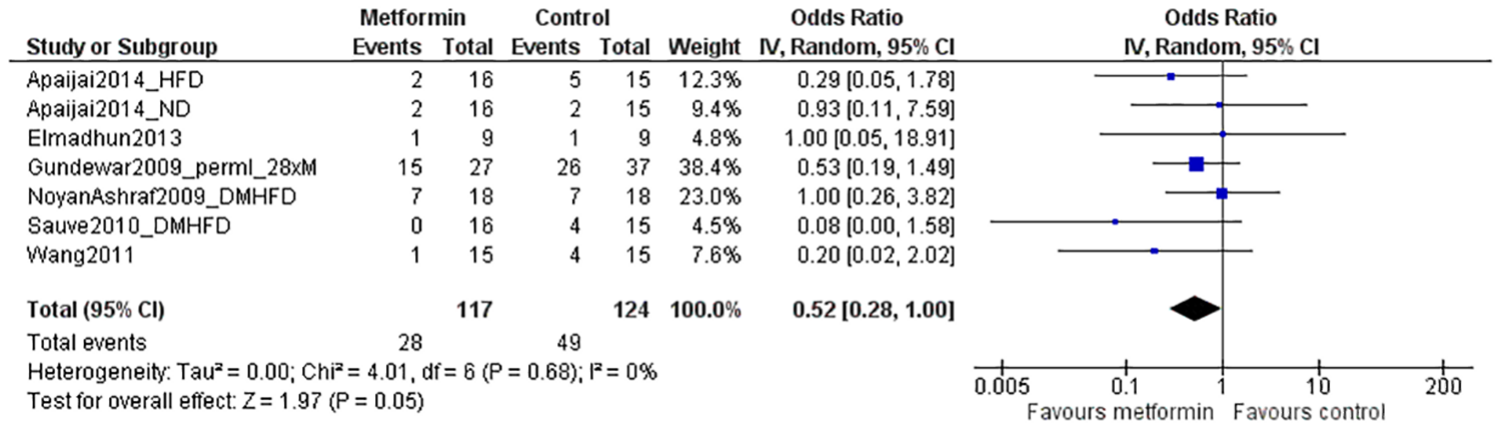
Forest plot of the effect of metformin on mortality after myocardial infarction *in vivo*. Outcome data from metformin-treated groups and control groups were extracted from primary studies and expressed as odds ratios (blue squares). The pooled effect estimate (diamond) indicates a borderline significant difference in mortality between metformin-treated animals and controls. SD = standard deviation, Total = sample size, CI = confidence interval, IV = inverse variance.

Mortality was reported in *in vivo* studies only. Data of seven comparisons from six studies could be included in the analysis, which were obtained from a total of 127 metformin-treated animals and 114 controls. The overall effect estimate indicated a borderline significant reduction in mortality after metformin treatment (OR 0.52[0.28, 1.00]; P = 0.05; Fig 7). There was low heterogeneity (0%; P = 0.68) in the overall analysis. Subgroup analysis for comorbidity showed no effect of the presence (OR 0.54[0.21, 1.43]) or absence (OR 0.51[0.21, 1.20]) of comorbidity on treatment efficacy (P = 0.92; figure E in S3 File). The effect of metformin did not reach significance in either of the subgroups, since the lower number of studies reduced the power in these groups. The same held true for subgroups based on timing of treatment (OR 0.56[0.22, 1.44] versus OR 0.48[0.20, 1.19]; P = 0.82; figure F in S3 File).

#### Sensitivity analyses

To test the robustness of our findings using the OR for binary outcomes, we reanalyzed the data using the RR. The overall effect of metformin on mortality was neutral (RR 0.75[0.54, 1.05]), as was the effect of comorbidity (RR 0.69[0.33, 1.44] in comorbid *versus* RR 0.77[0.52, 1.12] in healthy animals; P = 0.81). In contrast to the OR analysis, there was no difference between subgroups based on timing of treatment when using the RR (RR 0.71[0.35, 1.42] for treatment pre-ischemia *versus* RR 0.76[0.52, 1.12] for treatment during or after ischemia; P = 0.85).

Substituting the left ventricle/body weight outcome data from Inthachai2015 with the right ventricle/body weight or atrium/body weight data did not change the outcome of the meta-analysis for cardiac hypertrophy (SMD 0.33[-0.37, 1.04] *versus* 0.30[-0.41, 1.01] and 0.28[-0.43, 1.00], respectively).

#### Publication bias

We aimed to perform an analysis of publication bias based on funnel plots for the primary outcome measure IS/AAR%. However, because of the limited number of data points/comparisons (both for *ex vivo* and *in vivo* studies) and the fact that these data points were extracted from an even smaller number of studies (five *ex vivo* and seven *in vivo*), publication bias could not be reliably assessed and the analysis was no performed.

#### Descriptive summary of circulating troponin (secondary outcome)

Cardiac troponin was measured in one study[26], reporting that a single dose of metformin (125ug/kg) given at the onset of reperfusion led to a significant reduction in circulating troponin-T levels four hours after myocardial infarction.

## Discussion

Animal studies that reported beneficial effects of metformin on myocardial infarct size and postinfarction remodeling have recently instigated clinical trials aiming to reduce myocardial injury by the administration of metformin. Disappointingly, these studies reported no beneficial effect of metformin on myocardial damage during coronary bypass surgery, nor on postinfarction remodeling[4, 5]. Our current study is the first to systematically evaluate the effect of metformin on myocardial ischemia-reperfusion injury in animal models, in order to critically reappraise the scientific basis that fuelled the trials in patients.

Our meta-analysis (summarized in Table 4) of all preclinical studies reporting the effect of metformin administration on histological infarct size revealed that metformin significantly reduces myocardial infarct size, both *in vivo* and *ex vivo* in isolated heart preparations. With regard to the *in vivo* studies, we observed similar efficacy in healthy animals and in animals with comorbidities (diabetes, metabolic syndrome and/or insulin resistance), and with administration of metformin before ischemia or after the onset of ischemia (*i*.*e*. during the occlusion, or upon reperfusion).

**Table 4:** Summary of meta-analysis results.

Outcome	Model	(Sub)group	# studies	# comp	Effect of Met	Subgroup differences
IS/AAR%	*Ex vivo*	*Overall*	5	9	↓	
	*In vivo*	*Overall*	6	10	↓	
		*Comorbidity*				NS
		Yes	4	5	↓	
		No	6	8	↓	
		*Timing of Met*				NS
		Pre I	5	7	↓	
		During I / at R	4	6	↓	
LVEF	*In vivo*	*Overall*	8	12	↑	
		*Species*				NS
		Rat	4	6	↑	
		Mouse	4	6	↑	
		Duration of I*				NS
		Permanent	6	7	↑	
		Temporary	3	5	↑	
LVESD	*In vivo*	*Overall*	6	7	↓	
Hypertrophy	*In vivo*	*Overall*	6	8	=	
Mortality	*In vivo*	*Overall*	6	7	= /↑	
		*Comorbidity*				NS
		Yes	4	4	=	
		No	3	3	=	
		*Timing of Met*				NS
		Pre I	3	4	=	
		During I / at R	3	3	=	

Only subgroups eligible for analysis are shown. IS/AAR% = infarct size as percentage of area at risk, LVEF = left ventricular ejection fraction, LVESD = left ventricular end-systolic diameter, Met = metformin treatment, I = ischemia, R = reperfusion, comp = comparisons, ↓ = decrease in outcome, ↑ = increase in outcome, = no effect on outcome, NS = not significant,

*post-hoc analysis.

Consistent with its infarct size-limiting effect, treatment with metformin significantly improved left ventricular ejection fraction in our meta-analysis. Interestingly, a post-hoc subgroup analysis revealed that this beneficial effect was observed in models of temporary coronary artery ligation, as well as permanent ligation, suggesting a beneficial effect of metformin beyond mere infarct size limitation. In all but one [26] of these studies, metformin was administered for several weeks before coronary occlusion. Our analyses further show a positive effect of metformin on the LVESD, no effect of metformin on cardiac hypertrophy was observed, and a borderline significant effect on mortality. Importantly, the evidence for our secondary outcomes was limited to 7–11 comparisons, and the results should be interpreted with care. Of note, to obtain a complete overview of the evidence available we included data on mortality regardless of cause and timing. Since none of the individual studies showed any effect of metformin on mortality, the pooled effect estimate was barely significant and there was very low heterogeneity in the analysis (I^2^ = 0%) an analysis of the influence of cause and timeframe was not deemed feasible.

Overall, the beneficial effect of metformin on cardiac function seems consistent across outcome measures, with very few studies reporting adverse effects of metformin on any of the outcomes. However, some of our analysis showed substantial between-study heterogeneity, some of which remained unexplained after subgroup analysis. The internal validity may be an important source of this residual heterogeneity, but insufficient reporting prevented any analyses assessing this. Furthermore, heterogeneity may be partially due to experimental characteristics not investigated in the current review, such as rodent strain, anaesthetic type and metformin administration route.

In conclusion, we observed consistent cardioprotective effects of metformin, on histological infarct size, LVEF, as well as LVESD. Although subgroup analysis point to a superior effect of metformin after single dose administration, these results have to be interpreted with caution due to the limited number of studies per subgroup, and other differences in experimental design between the subgroups. Of note, as in any meta-analysis, the number of subgroup analyses that could reliably be performed in this review was limited by the (expected) amount of evidence available. Therefore, many of our proposed subgroup variables could not be investigated. These, and other possible sources of heterogeneity (e.g. the dose and formulation of metformin, the and the internal study validity) likely (partly) account for the residual heterogeneity in our analyses. Regarding the internal validity, our risk of bias assessment was hampered by the extremely poor reporting of possible sources of bias. Only 57% of studies reported randomization at any level of the experiment, 35% reported blinding, and none of the studies reported a sample size calculation. This is consistent with our previous findings in animal studies on the effect of ischemic conditioning in the heart[8] and the kidney[8, 37]. This might severely hamper the internal validity of the studies, since non-randomised and non-blinded animal studies[38] as well as clinical trials[39, 40] tend to overestimate treatment efficacy, compared to studies using appropriate randomization and blinding.

In addition to limitations in internal validity, the external validity of animal studies might be limited by several factors. First, there are important differences in the biology and pathophysiology between animals and humans[6]. We show that the present body of evidence has been predominantly obtained in rodents, and suggest that replication of these results in other (larger) animal species can increase the confidence in these findings. Secondly, the sex bias in this body of evidence (no studies reported using female animals) introduces indirectness, since the intention is to treat both male and female patients. Furthermore, the comorbidities and comedications that are present in patients, but not in most animal models, might severely interfere with the cardioprotective mechanism of metformin. Finally, it is important to critically compare the scenarios in which metformin is tested in animals and patients. Our meta-analysis reveals that most evidence points to a beneficial effect of metformin on infarct size in the setting of temporary occlusion of a coronary artery. This importantly differs from a scenario of chronic administration of metformin, commencing several hours after coronary reperfusion[5] and from the scenario of global myocardial ischemia in the setting of cardiac surgery with extracorporeal circulation[4]. Indeed, the two clinical studies that did focus on the effect of metformin on ischemia-reperfusion injury due to temporary coronary occlusion showed beneficial effects of metformin, but these studies have methodological shortcomings. In patients with an acute ST-elevation myocardial infarction, peak plasma concentrations of cardiac biomarkers were lower in patients with diabetes treated with metformin, but the retrospective study design is susceptible for bias[41]. In another study, the effect of metformin on myocardial ischemia-reperfusion injury was explored in patients with the metabolic syndrome who were scheduled for elective percutaneous coronary intervention for stable or unstable angina. Pretreatment with metformin (250 mg 3x/d for 7 days) reduced the occurrence of postprocedural myocardial damage[42]. However, this study has an open label design and involved only minor cardiac ischemic insults.

Finally, we were unable to assess the risk of publication bias in the present body of evidence, and therefore cannot exclude the possibility that such a bias is present, possibly leading to an overestimation of the treatment effect.

Based on our critical reappraisal of the preclinical evidence that metformin has direct cardioprotective effects, we would like to make two recommendations for further research. First, we would like to endorse the recommendations by Landis et al. that animal studies should at least provide information about randomization, blinding, sample size calculation and handling of data[43]. Secondly, we have not lost faith that metformin limits myocardial infarct size when it is administered immediately before coronary reperfusion in patients with an acute myocardial infarction. An adequately powered, confirmatory preclinical study should be performed, using proper randomisation and blinding, and optimized external validity (*e*.*g*. using comorbid animals of both sexes, and treatment regimes which are comparable to those in patients). Given the fact that metformin is cheap, worldwide available and that it’s (long term) safety is proven, we would like to encourage a randomized clinical trial in patients with an acute myocardial infarction in which metformin administration is given immediately at the moment of reperfusion.

## Supporting information

S1 File Systematic review protocol. (PDF)

S2 File Full search strategy. (PDF)

S3 File Supporting figures A-F. (PDF)

S4 File PRISMA checklist. (PDF)
