# Correction: A systematic review and meta-analysis of the protective effects of metformin in experimental myocardial infarction

**DOI:** 10.1371/journal.pone.0195858

**Published:** 2018-04-09

**Authors:** Nienke A. Hesen, Niels P. Riksen, Bart Aalders, Michelle A. E. Brouwer, Merel Ritskes-Hoitinga, Saloua El Messaoudi, Kimberley E. Wever

Dr. Michelle A. E. Brouwer should be included in the author byline. She should be listed as the fourth author, and her affiliation is: 1. SYstematic Review Centre for Laboratory animal Experimentation (SYRCLE), Department for Health Evidence, Nijmegen Institute for Health Sciences, Radboud University Medical Center, Nijmegen, The Netherlands. The contribution of this author are as follows: Data curation, Writing–review & editing.

The correct citation is: Hesen NA, Riksen NP, Aalders B, Brouwer MAE, Ritskes-Hoitinga M, El Messaoudi S, et al. (2017) A systematic review and meta-analysis of the protective effects of metformin in experimental myocardial infarction. PLoS ONE 12(8): e0183664. https://doi.org/10.1371/journal.pone.0183664
