# Supplementary material for: A systematic review and meta-analysis of the protective effects of metformin in experimental myocardial infarction
Source: PLoS One. 2017 Aug 23;12(8):e0183664. doi: 10.1371/journal.pone.0183664 (PMC5568412; doi:10.1371/journal.pone.0183664)
Supplement: S1 File — (PDF) [file pone.0183664.s001.pdf]

SYSTEMATIC REVIEW PROTOCOL FOR ANIMAL INTERVENTION STUDIES

FORMAT BY SYRCLE (WWW.SYRCLE.NL)
VERSION 2.0 (DECEMBER 2014)

Item #	Section/Subsection/Item	Description	Check for approval
	A. General		
1.	Title of the review	Effects of metformin on ischemic myocardial injury	
2.	Authors (names, affiliations, contributions)	KE Wever - SYRCLE, Radboudumc, The Netherlands – study concept and design, literature search, study selection, data extraction, data analysis, RoB assessment, manuscript writing, approval of manuscript<br><br>BF Aalders - SYRCLE and Internal Medicine, Radboudumc, The Netherlands - literature search, study selection, data extraction, data analysis, RoB assessment, approval of manuscript<br><br>NA Hesen - SYRCLE, Radboudumc, The Netherlands – study selection, data extraction, RoB assessment, manuscript writing, approval of manuscript<br><br>M Ritskes-Hoitinga, SYRCLE, Radboudumc, The Netherlands – funding, approval of manuscript<br><br>NP Riksen, Internal Medicine, Radboudumc, The Netherlands - study design, approval of manuscript<br><br>S El Messaoudi, Internal Medicine, Radboudumc, The Netherlands – study design, manuscript writing, approval of manuscript	
3.	Other contributors (names, affiliations, contributions)	A Tillema - Librarian, Radboudumc, The Netherlands – search strategy design	
4.	Contact person + e-mail address	KE Wever, kim.wever@radboudumc.nl	
5.	Funding sources/sponsors	None	
6.	Conflicts of interest	The authors have no conflicts of interest to declare	
7.	Date and location of protocol registration	20 October 2015, www.syrcle.nl	
8.	Registration number (if applicable)	NA	
9.	Stage of review at time of registration	Screening on title and abstract in progress	
	B. Objectives		
	Background		
10.	What is already known about this disease/model/intervention? Why is it important to do this review?	Morbidity and mortality in patients with an acute myocardial infarction remains high, in spite of major advances in prevention and treatment. An acute ischemic event can have devastating effects, and if a patient survives such an event, cardiac function is often complicated in a later stage by the development of heart failure. This holds true especially for patients with type 2 diabetes, who have a marked increased risk for coronary heart disease, and may even develop heart failure independent from coronary artery disease ('diabetic cardiomyopathy').<br><br>The biguanide compound metformin is a glucose lowering drug which has been commonly used to lower glucose levels in patients since the 1990s. Between 1998 and 2011, a number of cohort studies have indicated that in type 2 diabetic patients, treatment with metformin is associated with a lower	

		cardiovascular morbidity and mortality, compared with alternative glucoselowering drugs. These observations suggest that metformin exerts direct protective effects on the heart, independent of its glucose-lowering action.<br><br>A number of animal studies (mostly performed between 2002 and 2011) indeed show protective effects of metformin in animal models of myocardial ischaemia-reperfusion injury and cardiac remodeling. However, recent randomized clinical trials have shown no effect of metformin on cardiovascular outcomes in non-diabetic patients after myocardial infarction or CABG. This raises the question why the protective effects of metformin appear to have translated from bedside to bench, but not back to bedside. The preclinical evidence has not yet been systematically reviewed. The internal and external validity of the preclinical studies, as well as possible publication bias, may have influenced the translational value of the animal studies. We aim to address these matters in the present systematic review.	
	Research question		
11.	Specify the disease/health problem of interest	Ischemic myocardial injury, due to coronary occlusion	
12.	Specify the population/species studied	Animals hearts *in vivo* or *in vitro*.	
13.	Specify the intervention/exposure	Metformin	
14.	Specify the control population	Non-treated or vehicle-treated hearts	
15.	Specify the outcome measures	Ischemic myocardial injury, as measured by myocardial structure, function or injury markers	
16.	State your research question (based on items 11-15)	What is the effect of metformin on ischemic myocardial injury in animals (or animal hearts) undergoing cardiac ischemia, when compared to non-treated or vehicle-treated animals?	
	C. Methods		
	Search and study identification		
17.	Identify literature databases to search (*e.g.* Pubmed, Embase, Web of science)	X MEDLINE via PubMed ☐Web of Science<br>☐SCOPUS X EMBASE<br>☐Other, namely:<br>☐Specific journal(s), namely:	
18.	Define electronic search strategies (*e.g.* use the step by step search guide[15] and animal search filters[20, 21])	When available, please add a supplementary file containing your search strategy: [see last page of this protocol]	
19.	Identify other sources for study identification	X Reference lists of included studies ☐Books<br>X Reference lists of relevant reviews<br>☐Conference proceedings, namely:<br>☐Contacting authors/ organisations, namely:<br>☐Other, namely:	
20.	Define search strategy for these other sources	One reviewer will scan reference lists for eligible references. Subsequently, these papers will be assessed full-text by two independent reviewers.	
	Study selection		
21.	Define screening phases (*e.g.* pre-screening based on title/abstract, full text screening, both)	Phase 1: screening on title and abstract<br>Phase 2: full text screening	
22.	Specify (a) the number of reviewers per screening phase and (b) how discrepancies will be resolved	At least 2 screeners per screening phase.<br>Discrepancies will be resolved by discussion.	

	Define all inclusion and exclusion criteria based on:		
23.	Type of study (design)	Inclusion criteria: studies including a control group undergoing no treatment or vehicle treatment *versus* a metformin-treated group<br>Exclusion criteria: studies without a suitable control group.	
24.	Type of animals/population (*e.g.* age, gender, disease model)	Inclusion criteria: all animal species with or without cardiovascular comorbidities, subjected to cardiac ischemia *in vivo* or *in vitro*. Possible cardiac ischemia models include: (transient or permanent) coronary occlusion, aortic constriction and cardiac transplantation.<br><br>Exclusion criteria: non-ischemic myocardial damage (e.g. , aorta-caval fistula-induced volume overload, diabetic cardiomyopathy, pulmonary banding induced overload, cardiac pacing, isoproterenol induced HF), studies using only genetically modified (KO or KI) animals, studies using animals with comorbidity not related to cardiovascular disease.	
25.	Type of intervention (*e.g.* dosage, timing, frequency)	Inclusion criteria: treatment with metformin in any dose, formulation and route of administration (*e.g.* LV-injection, oral administration, perfusion, i.v. injection etc.)<br>Exclusion criteria: none	
26.	Outcome measures	Inclusion criteria: cardiovascular outcome measures related to cardiac function or ischemic cardiac injury.<br>Exclusion criteria: no relevant OMs reported	
27.	Language restrictions	Inclusion criteria: all languages<br>Exclusion criteria: none	
28.	Publication date restrictions	Inclusion criteria: all years of publication<br>Exclusion criteria: none	
29.	Other	Inclusion criteria: none<br>Exclusion criteria: co-interventions and co-medication with any drug/treatment other than analgesia or anaesthesia.	
30.	Sort and prioritize your exclusion criteria per selection phase	Selection phase: title and abstract<br>1. no original full paper containing data<br>2. not an in or ex vivo animal study<br>3. no ischemic heart disease model (through occlusion)<br>4. not on metformin<br><br>Selection phase: full-text screening<br>All of the above, with addition of:<br>5. full text unretrievable<br>6. no cardiovascular outcome measures<br>7. genetically modified animals only<br>8. unsuitable co-morbidity or co-intervention	
	Study characteristics to be extracted (for assessment of external validity, reporting quality)		
31.	Study ID (*e.g.* authors, year)	First author, title, year of publication	
32.	Study design characteristics (*e.g.* experimental groups, number of animals)	Experimental groups, control group(s), number of animals per group	
33.	Animal model characteristics (*e.g.* species, gender, disease induction)	Species, sex, weight, age, co-morbidity, in vivo/in vitro model, anaesthesia, method of induction of cardiac ischemia, duration of ischemia, duration of reperfusion (if applicable)	
34.	Intervention characteristics (*e.g.* intervention, timing, duration)	Dose, timing of administration, route of administration	

35.	Outcome measures	Selected outcomes for meta-analysis:<br>1. IS/AAR % (primary OM)<br><br>Secondary OMs:<br>1. (HS) Troponin I<br>2. LVEF<br>3. LVESD<br>4. Cardiac hypertrophy<br>5. Mortality<br><br>For all outcome measures, we will extract or recalculate the mean, SD and number of animals per group in the experimental and control group(s).	
36.	Other (*e.g.* drop-outs)	Number and reason	
	Assessment risk of bias (internal validity) or study quality		
37.	Specify (a) the number of reviewers assessing the risk of bias/study quality in each study and (b) how discrepancies will be resolved	At least two reviewers will assess the risk of bias and study quality of all studies reporting on one of the outcome measures selected for meta-analysis.<br>Discrepancies will be resolved by discussion.	
38.	Define criteria to assess (a) the internal validity of included studies (*e.g.* selection, performance, detection and attrition bias) and/or (b) other study quality measures (*e.g.* reporting quality, power)	☐By use of SYRCLE's Risk of Bias tool[4]<br>X By use of SYRCLE's Risk of Bias tool, adapted as follows: additional scoring of reporting of study quality indicators "reporting of any randomisation", "reporting of any blinding" , "reporting of temperature regulation", "reporting of a power calculation" and "reporting of a conflict of interest statement".<br>☐By use of CAMARADES' study quality checklist, e.g [22]<br>☐By use of CAMARADES' study quality checklist, adapted as follows:<br>☐Other criteria, namely:	
	Collection of outcome data		
39.	For each outcome measure, define the type of data to be extracted (*e.g.* continuous/dichotomous, unit of measurement)	• IS/AAR %, continuous in %<br>• (HS) Troponin I, continuous in ng/ml<br>• LVEF, continuous in %<br>• LVESD, continuous in mm3<br>• Mortality, incidence<br>• Cardiac hypertrophy (various units of measurement possible, method of extraction to be determined)	
40.	Methods for data extraction/retrieval (*e.g.* first extraction from graphs using a digital screen ruler, then contacting authors)	1. Direct extraction of data from tables, text and figures<br>2. Extraction from graphs using digital screen ruler<br>3. Contacting authors by e-mail for original data if data not reported or unclear<br><br>All data will be collected as mean and standard deviation (SD). Standard error of the mean will be recalculated to SD. In case the number of animals is unclear and cannot be retrieved, a conservative estimate will be made. In case the data are reported as median and interquartile range, the authors will be contacted for raw data. In case an outcome was measured at multiple time points, the measurement of greatest efficacy will be chosen. Multiple treatment regimes from one study will be extracted as separate comparisons.<br><br>In case of missing data and no author contact details, or	

		no response from authors within 3 weeks including a reminder, the study will be omitted from analysis.	
41.	Specify (a) the number of reviewers extracting data and (b) how discrepancies will be resolved	One reviewer will extract the data, a second reviewer will check the extracted data for inconsistencies.	
	Data analysis/synthesis		
42.	Specify (per outcome measure) how you are planning to combine/compare the data (*e.g.* descriptive summary, meta-analysis)	Meta-analysis will be performed for all selected outcomes reported in three or more studies, but in case of high heterogeneity studies will not be pooled. If less than three studies report on a selected outcome, a descriptive summary will be provided.	
43.	Specify (per outcome measure) how it will be decided whether a meta-analysis will be performed	A meta-analysis will be performed if ≥3 studies report on a specific outcome measure. For subgroup analysis a minimum of 3 studies per subgroup is required.	
	If a meta-analysis seems feasible/sensible, specify (for each outcome measure):		
44.	The effect measure to be used (*e.g.* mean difference, standardized mean difference, risk ratio, odds ratio)	For IS/AAR and LVEF, the raw difference in means will be used, since these are relative outcome measures expressed as a %. For troponin and LVESD, we aim to use a normalized mean difference, if there are sham or baseline data available for the selected outcome measures. If (for any of the OMs) such data are not reported in the majority of studies, we aim to use a standardized mean difference. For mortality, an odds ratio will be calculated.	
45.	The statistical model of analysis (*e.g.* random or fixed effects model)	Random effects model for all outcome measures	
46.	The statistical methods to assess heterogeneity (*e.g.* I^2, Q)	(residual) I^2 and adjusted R^2 for all outcome measures	
47.	Which study characteristics will be examined as potential source of heterogeneity (subgroup analysis)	- Animal species (stratified per species)<br>- Sex (stratified m vs f vs mixed vs not reported)<br>- Dose of metformin (linear or stratified)<br>- Timing of metformin treatment (linear or stratified)<br>- Co-morbidity (stratified y/n)<br>- Injury model (stratified by type, e.g. in vitro vs in vivo)<br>- reporting of randomisation (stratified y/n)<br>- reporting of blinding (stratified y/n)	
48.	Any sensitivity analyses you propose to perform	Choose 1 specific time-point for outcome measure, instead of choosing the time-point of greatest efficacy. Perform SMD analysis instead of MD or NMD if applicable.	
49.	Other details meta-analysis (*e.g.* correction for multiple testing, correction for multiple use of control group)	We will perform a Holm-Bonferroni correction on the p-value, depending on the number of subgroup analyses performed.<br>Correction for multiple use of control group will be performed by dividing the number of animals in the control group by the number of comparisons performed with this control group.	
50.	The method for assessment of publication bias	Produce funnel plots and perform visual analysis of these plots. We are aware that funnel plots of SMD are susceptible to distortion and will omit the assessment of publication bias if this is suspected for our dataset. In addition, we aim to perform Trim and Fill analysis and Egger's test for small study effects for outcome measures containing 20+ studies	
