# Supplementary material for: A systematic review and meta-analysis of the protective effects of metformin in experimental myocardial infarction
Source: PLoS One. 2017 Aug 23;12(8):e0183664. doi: 10.1371/journal.pone.0183664 (PMC5568412; doi:10.1371/journal.pone.0183664)
Supplement: S2 File — (PDF) [file pone.0183664.s002.pdf]

	Systematic searches (performed on 12 May 2017)
PubMed	
Metformin	metformin[MH] OR biguanides[MH:noExp] OR metformin*[TW] OR biguanid*[TW] OR dimethylbiguanidine[TW] OR dimethylguanylguanidine[TW] OR glucophage[TW]
Heart	heart[MH] OR myocardium[MH] OR coronary occlusion[MH] OR heart injuries[MH] OR heart transplantation[MH] OR heart failure[MH] OR myocardial ischemia[MH] OR heart[TW] OR hearts[TW] OR cardiac[TW] OR myocardial[TW] OR myocardium[TW] OR myocardia[TW] OR myocard[TW] OR cardioprotection[TW] OR cardioprotective[TW] OR cardio-protection[TW] OR cardio-protective[TW] OR coronary[TW]
(heart and) Ischemia	heart injuries[MH] OR cardiovascular diseases[MH] OR heart disease[MH] OR ischemia[MH] OR infarction[MH] OR warm ischemia[MH] OR cold ischemia[MH] OR reperfusion injury[MH] OR myocardial ischemia[MH] OR coronary occlusion[MH] OR ischemia[TW] OR ischaemia[TW] OR ischemic[TW] OR ischaemic[TW] OR infarct[TW] OR infarcts[TW] OR infarction[TW] OR infarctions[TW] OR infarcted[TW] OR reperfusion injury[TW] OR reperfusion injuries[TW] OR I/R[TW] OR IRI[TW] OR occlusion[TW] OR transplantation[MH] OR heart transplantation[MH] OR primary graft dysfunction[MH] OR graft survival[MH] OR graft rejection [MH] OR transplants[MH] OR transplantation[TW] OR transplantations[TW] OR transplant[TW] OR transplants[TW] OR graft[TW] OR grafts[TW]OR heart failure[MH] OR failure[TW] OR decompensation[TW] OR insufficiency[TW] OR incompetence[TW] OR overload[TW] OR cardiac function[TW] OR cardiac functions[TW] OR cardiac fibrosis[TW]
Animals	Search filter [11]
EMBASE	
Metformin	exp metformin/ or (metformin* or biguanid* or dimethylbiguanidine or dimethylguanylguanidine or glucophage).ti,ab,kw.
Heart	exp heart/ or exp coronary artery occlusion/ or exp heart injury/ or exp myocardial disease/ or ischemic heart disease/ or heart failure/ or exp heart transplantation/ or (heart or hearts or cardiac or myocardial or myocardium or myocardia or myocard or cardioprotection or cardioprotective or cardio-protection or cardio-protective or coronary).ti,ab,kw.
(heart and) Ischemia	exp heart injury/ or exp myocardial disease/ or exp ischemic heart disease/ or exp ischemia/or exp infarction/ or exp reperfusion injury/ or exp ischemic heart disease/ or exp coronary artery occlusion/ or exp graft dysfunction/ or exp transplantation/ or exp heart transplantation/ or exp heart failure/ or exp heart muscle fibrosis/ or exp heart function/ or (ischemia or ischaemia or ischemic or ischaemic or infarct or infarcts or infarction or infarctions or infarcted or reperfusion injury or reperfusion injuries or I/R or IRI or occlusion or transplantation or transplantations or transplant or transplants or graft or grafts or failure or decompensation or insufficiency or incompetence or overload or cardiac function or cardiac functions or cardiac fibrosis).ti,ab,kw.
Animals	Search filter [10]
