# Supplementary material for: A systematic review and meta-analysis of the protective effects of metformin in experimental myocardial infarction
Source: PLoS One. 2017 Aug 23;12(8):e0183664. doi: 10.1371/journal.pone.0183664 (PMC5568412; doi:10.1371/journal.pone.0183664)
Supplement: S3 File — (PDF) [file pone.0183664.s003.pdf]

Study or Subgroup	Metformin Mean [IS/AAR%]	Metformin SD [IS/AAR%]	Metformin Total	Control Mean [IS/AAR%]	Control SD [IS/AAR%]	Control Total	Weight	Mean Difference IV, Random, 95% CI [IS/AAR%]
**1.3.3 Comorbidity**								
Apaijai2014_HFD	20.8	4.6	6	33.2	5.6	6	11.0%	-12.40 [-18.20, -6.60]
Calvert2008_DM_atR	56	6.1	9	67.3	8.3	6	10.2%	-11.30 [-19.05, -3.55]
Calvert2008_DM_preI	44.1	16.7	8	67.3	8.3	6	7.6%	-23.20 [-36.54, -9.86]
Oidor-Chan2016_DM	11.6	7.7	4	27.1	6.3	4	9.3%	-15.50 [-25.25, -5.75]
**Subtotal (95% CI)**			**27**			**22**	**38.1%**	**-13.60 [-17.60, -9.60]**
Heterogeneity: Tau² = 0.00; Chi² = 2.64, df = 3 (P = 0.45); I² = 0%								
Test for overall effect: Z = 6.67 (P < 0.00001)								
**1.3.4 Healthy**								
Apaijai2014_ND	18.7	6.1	6	24.7	4.1	6	11.0%	-6.00 [-11.88, -0.12]
Calvert2008_healthy_atR	25.9	9.5	12	50.6	4.2	12	11.0%	-24.70 [-30.58, -18.82]
Calvert2008_healthy_preI	19.4	6.7	12	50.6	4.2	12	11.5%	-31.20 [-35.67, -26.73]
Gundewar2009_60I_1xM	48.9	7.9	10	68.6	7.4	10	10.6%	-19.70 [-26.41, -12.99]
Oidor-Chan2016_healthy	6.3	4.1	4	19.3	4.3	4	11.0%	-13.00 [-18.82, -7.18]
Paiva2009_in vivo	34	17	8	62	13.2	7	6.8%	-28.00 [-43.31, -12.69]
**Subtotal (95% CI)**			**52**			**51**	**61.9%**	**-20.07 [-28.63, -11.52]**
Heterogeneity: Tau² = 99.42; Chi² = 54.62, df = 5 (P < 0.00001); I² = 91%								
Test for overall effect: Z = 4.60 (P < 0.00001)								
**Total (95% CI)**			**79**			**73**	**100.0%**	**-18.11 [-24.09, -12.14]**
Heterogeneity: Tau² = 75.65; Chi² = 65.98, df = 9 (P < 0.00001); I² = 86%								
Test for overall effect: Z = 5.94 (P < 0.00001)								
Test for subgroup differences: Chi² = 1.80, df = 1 (P = 0.18), I² = 44.6%								

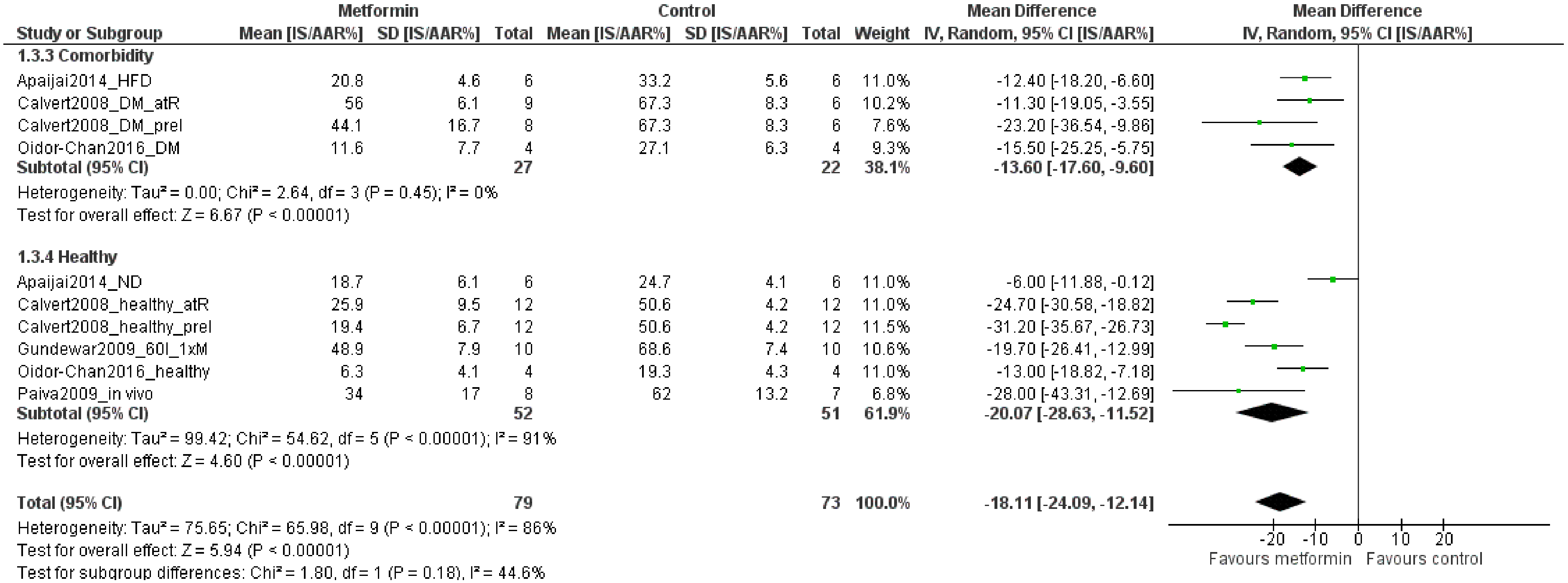

Figure A: Forest plot of the effect of metformin on myocardial infarct size, stratified by comorbidity (yes/no). Infarct size data expressed as a percentage of the area at risk (IS/AAR%) in metformin-treated groups and control groups were extracted from primary studies and expressed as mean differences (green squares). The pooled effect estimates (diamonds) show a significant reduction of infarct size in metformin-treated animals, both in healthy and comorbid animals. There was no difference in treatment efficacy between the subgroups. SD = standard deviation, Total = sample size, CI = confidence interval, IV = inverse variance, HFD = high fat diet, ND = normal diet, DM = diabetes mellitus, 60I = 60 minutes of ischemia, 1xM = single dose of metformin, atR = treatment at reperfusion, preI = treatment pre-ischemia.

Study or Subgroup	Metformin Mean [IS/AAR%]	Metformin SD [IS/AAR%]	Metformin Total	Control Mean [IS/AAR%]	Control SD [IS/AAR%]	Control Total	Weight	Mean Difference IV, Random, 95% CI [IS/AAR%]
**1.4.7 Metformin pre I**								
Apaijai2014_HFD	20.8	4.6	6	33.2	5.6	6	11.0%	-12.40 [-18.20, -6.60]
Apaijai2014_ND	18.7	6.1	6	24.7	4.1	6	11.0%	-6.00 [-11.88, -0.12]
Calvert2008_DM_preI	44.1	16.7	8	67.3	8.3	6	7.6%	-23.20 [-36.54, -9.86]
Calvert2008_healthy_preI	19.4	6.7	12	50.6	4.2	12	11.5%	-31.20 [-35.67, -26.73]
Oidor-Chan2016_DM	11.6	7.7	4	27.1	6.3	4	9.3%	-15.50 [-25.25, -5.75]
Oidor-Chan2016_healthy	6.3	4.1	4	19.3	4.3	4	11.0%	-13.00 [-18.82, -7.18]
**Subtotal (95% CI)**			**40**			**38**	**61.4%**	**-16.73 [-25.76, -7.69]**
Heterogeneity: Tau² = 111.79; Chi² = 56.68, df = 5 (P < 0.00001); I² = 91%								
Test for overall effect: Z = 3.63 (P = 0.0003)								
**1.4.8 Metformin during I / at R**								
Calvert2008_DM_atR	56	6.1	9	67.3	8.3	6	10.2%	-11.30 [-19.05, -3.55]
Calvert2008_healthy_atR	25.9	9.5	12	50.6	4.2	12	11.0%	-24.70 [-30.58, -18.82]
Gundewar2009_60I_1xM	48.9	7.9	10	68.6	7.4	10	10.6%	-19.70 [-26.41, -12.99]
Paiva2009_in vivo	34	17	8	62	13.2	7	6.8%	-28.00 [-43.31, -12.69]
**Subtotal (95% CI)**			**39**			**35**	**38.6%**	**-20.12 [-26.79, -13.44]**
Heterogeneity: Tau² = 28.08; Chi² = 8.34, df = 3 (P = 0.04); I² = 64%								
Test for overall effect: Z = 5.91 (P < 0.00001)								
**Total (95% CI)**			**79**			**73**	**100.0%**	**-18.11 [-24.09, -12.14]**
Heterogeneity: Tau² = 75.65; Chi² = 65.98, df = 9 (P < 0.00001); I² = 86%								
Test for overall effect: Z = 5.94 (P < 0.00001)								
Test for subgroup differences: Chi² = 0.35, df = 1 (P = 0.55), I² = 0%								

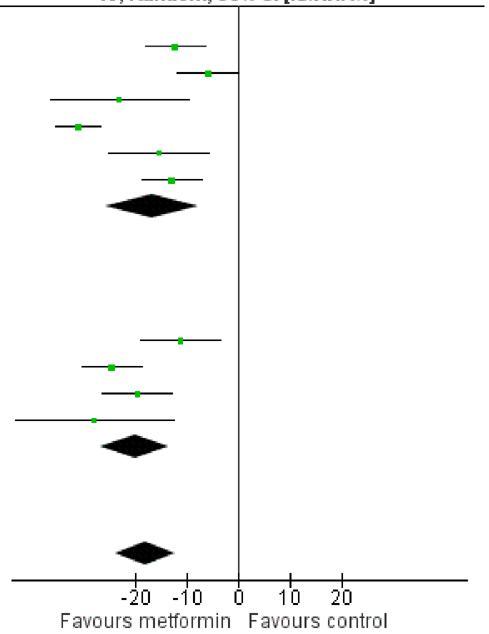

Figure B: Forest plot of the effect of metformin on myocardial infarct size, stratified by timing of administration. Infarct size data expressed as a percentage of the area at risk (IS/AAR%) in metformin-treated groups and control groups were extracted from primary studies and expressed as mean differences (green squares). The pooled effect estimates (diamonds) show a significant reduction of infarct size in animals treated with metformin before ischemia (pre I), as well as during ischemia or reperfusion (during I / at R). There was no difference in treatment efficacy between the subgroups. SD = standard deviation, Total = sample size, CI = confidence interval, IV = inverse variance, HFD = high fat diet, ND = normal diet, DM = diabetes mellitus, 60I = 60 minutes of ischemia, 1xM = single dose of metformin, atR = treatment at reperfusion, preI = treatment pre-ischemia.

Study or Subgroup	Metformin Mean [LVEF]	Metformin SD [LVEF]	Metformin Total	Control Mean [LVEF]	Control SD [LVEF]	Control Total	Weight	Mean Difference IV, Random, 95% CI [LVEF]
**1.8.1 Mouse**								
Calvert2008_healthy_tNR	60	7	9	44.3	10.7	8	8.5%	15.70 [6.99, 24.41]
Gundewar2009_60I_1xM	29.5	16.3	18	24.9	6.7	10	8.7%	4.60 [-4.00, 13.20]
Gundewar2009_60I_28xM	35.5	11.1	18	24.9	6.7	10	10.7%	10.60 [4.00, 17.20]
Gundewar2009_permI_28xM	28.8	14.1	12	32.4	18.5	11	5.1%	-3.60 [-17.13, 9.93]
Sun2017	41.6	3.9	10	35.2	5	10	13.7%	6.40 [2.47, 10.33]
Yue2016	34	6.1	8	26.1	5.5	8	11.7%	7.90 [2.21, 13.59]
**Subtotal (95% CI)**			**75**			**57**	**58.5%**	**7.79 [4.34, 11.24]**
Heterogeneity: Tau² = 6.08; Chi² = 7.58, df = 5 (P = 0.18); I² = 34%								
Test for overall effect: Z = 4.42 (P < 0.00001)								
**1.8.2 Rat**								
Apaijai2014_HFD	66	9.4	11	52.3	8.8	11	9.6%	13.70 [6.09, 21.31]
Apaijai2014_ND	69.6	10.7	11	70.3	17	11	6.1%	-0.70 [-12.57, 11.17]
Apaijai2016_HFD	48.3	11.5	6	27.2	5.9	6	7.2%	21.10 [10.76, 31.44]
Apaijai2016_ND	35.7	6.4	6	36.2	5.6	6	10.5%	-0.50 [-7.30, 6.30]
Wang2011	52.4	24.3	14	37.6	25.9	11	2.9%	14.80 [-5.11, 34.71]
Yin2011	65.8	11.1	9	48.6	15.8	8	5.3%	17.20 [4.07, 30.33]
**Subtotal (95% CI)**			**57**			**53**	**41.5%**	**10.34 [2.11, 18.58]**
Heterogeneity: Tau² = 72.41; Chi² = 18.58, df = 5 (P = 0.002); I² = 73%								
Test for overall effect: Z = 2.46 (P = 0.01)								
**Total (95% CI)**			**132**			**110**	**100.0%**	**8.57 [4.87, 12.27]**
Heterogeneity: Tau² = 22.01; Chi² = 26.37, df = 11 (P = 0.006); I² = 58%								
Test for overall effect: Z = 4.54 (P < 0.00001)								
Test for subgroup differences: Chi² = 0.31, df = 1 (P = 0.58), I² = 0%								

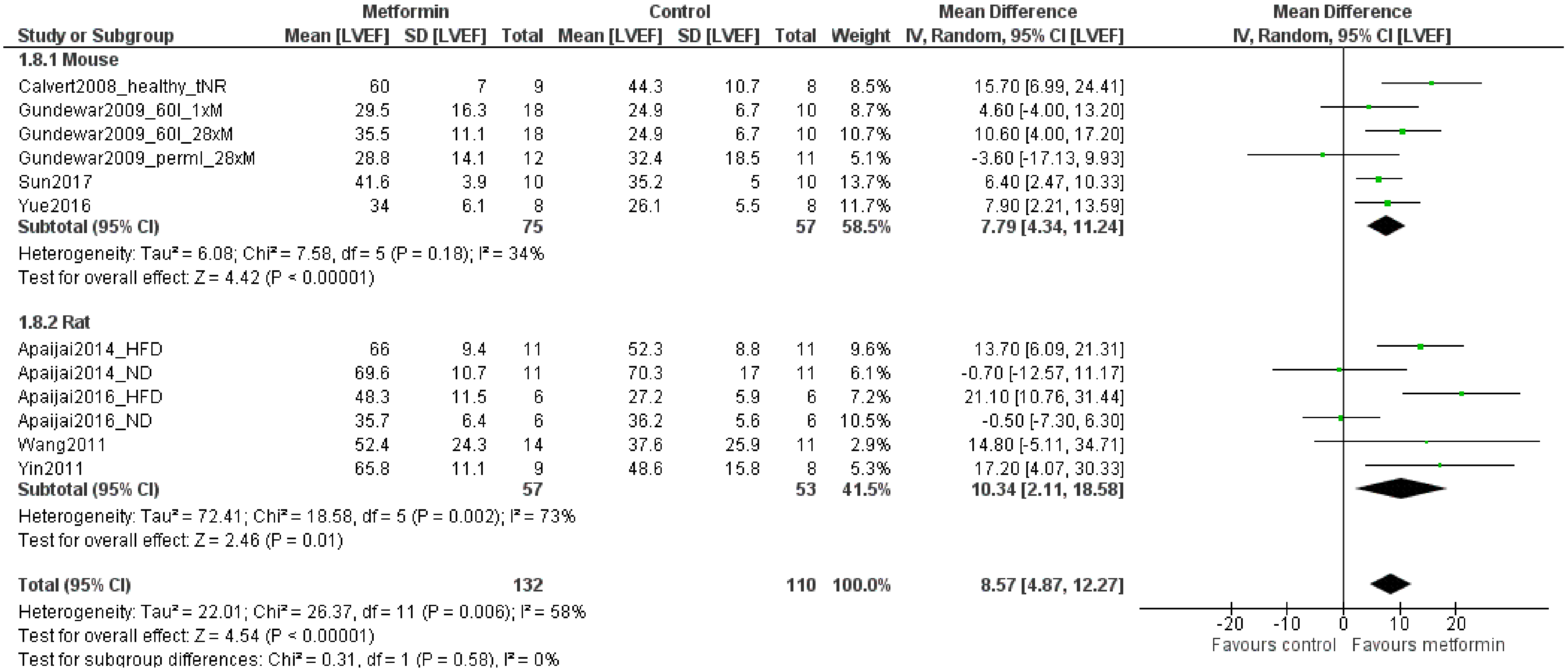

Figure C: Forest plot of the effect of metformin on left ventricular ejection fraction (LVEF) after myocardial infarction *in vivo*, stratified by species. Outcome data from metformin-treated groups and control groups were extracted from primary studies and expressed as mean differences (green squares). The bottom pooled effect estimate (diamond) shows a significant increase in the LVEF in metformin-treated animals. Metformin improved the LVEF in both mice and rats, and there was no significant difference between the subgroups. SD = standard deviation, Total = sample size, CI = confidence interval, IV = inverse variance, HFD = high fat diet, ND = normal diet, permI = permanent ischemia, 60I = 60 minutes of ischemia, 1xM = single dose of metformin, 28xM = 28 doses of metformin in total, tNR = time of treatment not reported.

	Metformin			Control				Mean Difference
Study or Subgroup	Mean [LVEF]	SD [LVEF]	Total	Mean [LVEF]	SD [LVEF]	Total	Weight	IV, Random, 95% CI [LVEF]
**1.9.1 permanent ischemia**								
Apaijai2016_HFD	48.3	11.5	6	27.2	5.9	6	7.2%	21.10 [10.76, 31.44]
Apaijai2016_ND	35.7	6.4	6	36.2	5.6	6	10.5%	-0.50 [-7.30, 6.30]
Gundewar2009_permI_28xM	28.8	14.1	12	32.4	18.5	11	5.1%	-3.60 [-17.13, 9.93]
Sun2017	41.6	3.9	10	35.2	5	10	13.7%	6.40 [2.47, 10.33]
Wang2011	52.4	24.3	14	37.6	25.9	11	2.9%	14.80 [-5.11, 34.71]
Yin2011	65.8	11.1	9	48.6	15.8	8	5.3%	17.20 [4.07, 30.33]
Yue2016	34	6.1	8	26.1	5.5	8	11.7%	7.90 [2.21, 13.59]
**Subtotal (95% CI)**			**65**			**60**	**56.4%**	**7.99 [2.58, 13.39]**
Heterogeneity: Tau² = 29.50; Chi² = 17.25, df = 6 (P = 0.008); I² = 65%								
Test for overall effect: Z = 2.90 (P = 0.004)								
**1.9.2 temporary ischemia**								
Apaijai2014_HFD	66	9.4	11	52.3	8.8	11	9.6%	13.70 [6.09, 21.31]
Apaijai2014_ND	69.6	10.7	11	70.3	17	11	6.1%	-0.70 [-12.57, 11.17]
Calvert2008_healthy_tNR	60	7	9	44.3	10.7	8	8.5%	15.70 [6.99, 24.41]
Gundewar2009_60I_1xM	29.5	16.3	18	24.9	6.7	10	8.7%	4.60 [-4.00, 13.20]
Gundewar2009_60I_28xM	35.5	11.1	18	24.9	6.7	10	10.7%	10.60 [4.00, 17.20]
**Subtotal (95% CI)**			**67**			**50**	**43.6%**	**9.64 [4.58, 14.69]**
Heterogeneity: Tau² = 14.61; Chi² = 7.22, df = 4 (P = 0.12); I² = 45%								
Test for overall effect: Z = 3.74 (P = 0.0002)								
**Total (95% CI)**			**132**			**110**	**100.0%**	**8.57 [4.87, 12.27]**
Heterogeneity: Tau² = 22.01; Chi² = 26.37, df = 11 (P = 0.006); I² = 58%								
Test for overall effect: Z = 4.54 (P < 0.00001)								
Test for subgroup differences: Chi² = 0.19, df = 1 (P = 0.66), I² = 0%								

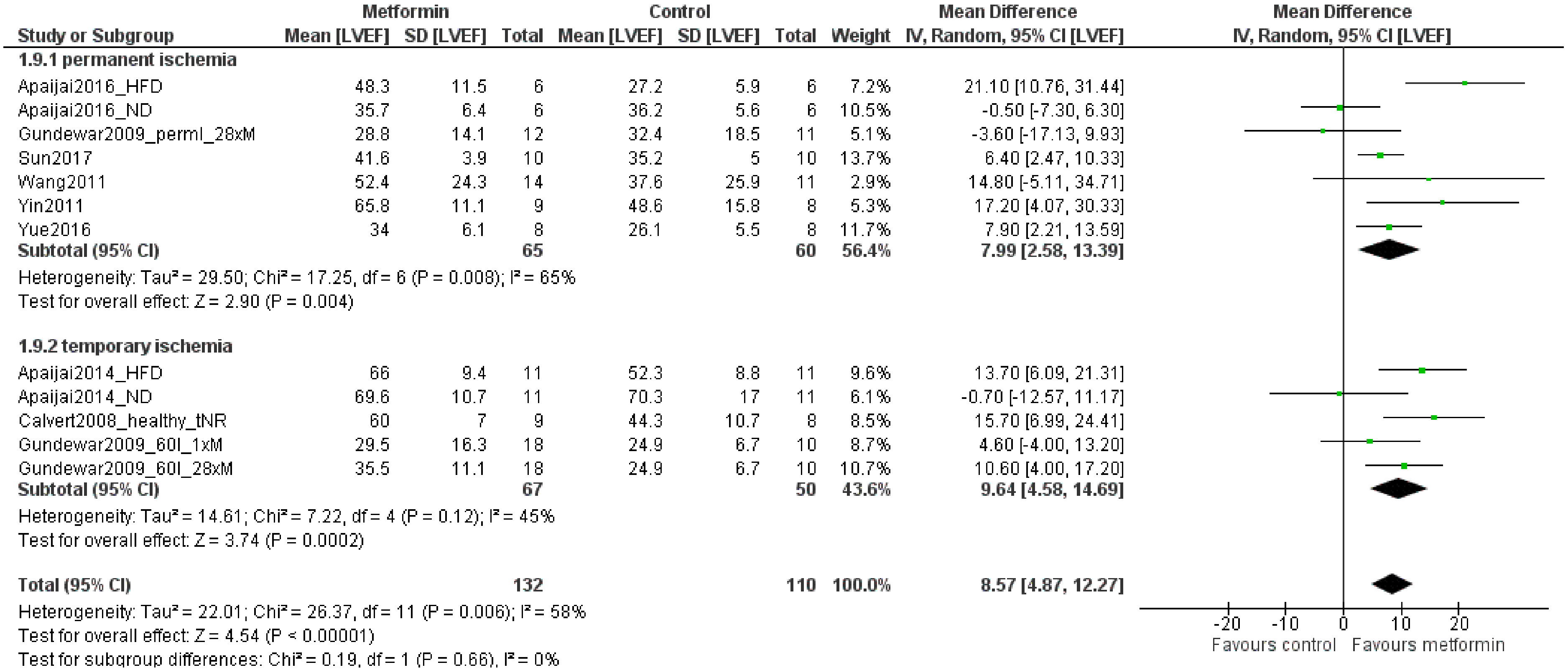

Figure D: Forest plot of the effect of metformin on left ventricular ejection fraction (LVEF) after myocardial infarction *in vivo*, stratified by occlusion model. Outcome data from metformin-treated groups and control groups were extracted from primary studies and expressed as mean differences (green squares). The bottom pooled effect estimate (diamond) shows a significant increase in the LVEF in metformin-treated animals. Metformin improved the LVEF in both permanent and temporary occlusion models, and there was no significant difference between the subgroups. SD = standard deviation, Total = sample size, CI = confidence interval, IV = inverse variance, HFD = high fat diet, ND = normal diet, permI = permanent ischemia, 60I = 60 minutes of ischemia, 1xM = single dose of metformin, 28xM = 28 doses of metformin in total, tNR = time of treatment not reported.

Study or Subgroup	Metformin Events	Metformin Total	Control Events	Control Total	Weight	Odds Ratio IV, Random, 95% CI
**1.11.3 Comorbidity**						
Apaijai2014_HFD	2	16	5	15	12.3%	0.29 [0.05, 1.78]
Elmadhun2013	1	9	1	9	4.8%	1.00 [0.05, 18.91]
NoyanAshraf2009_DMHFD	7	18	7	18	23.0%	1.00 [0.26, 3.82]
Sauve2010_DMHFD	0	16	4	15	4.5%	0.08 [0.00, 1.58]
**Subtotal (95% CI)**		**59**		**57**	**44.6%**	**0.54 [0.21, 1.43]**
Total events	10		17			
Heterogeneity: Tau² = 0.01; Chi² = 3.04, df = 3 (P = 0.39); I² = 1%						
Test for overall effect: Z = 1.23 (P = 0.22)						
**1.11.4 Healthy**						
Apaijai2014_ND	2	16	2	15	9.4%	0.93 [0.11, 7.59]
Gundewar2009_permI_28xM	15	27	26	37	38.4%	0.53 [0.19, 1.49]
Wang2011	1	15	4	15	7.6%	0.20 [0.02, 2.02]
**Subtotal (95% CI)**		**58**		**67**	**55.4%**	**0.51 [0.21, 1.20]**
Total events	18		32			
Heterogeneity: Tau² = 0.00; Chi² = 0.96, df = 2 (P = 0.62); I² = 0%						
Test for overall effect: Z = 1.54 (P = 0.12)						
**Total (95% CI)**		**117**		**124**	**100.0%**	**0.52 [0.28, 1.00]**
Total events	28		49			
Heterogeneity: Tau² = 0.00; Chi² = 4.01, df = 6 (P = 0.68); I² = 0%						
Test for overall effect: Z = 1.97 (P = 0.05)						
Test for subgroup differences: Chi² = 0.01, df = 1 (P = 0.92), I² = 0%						

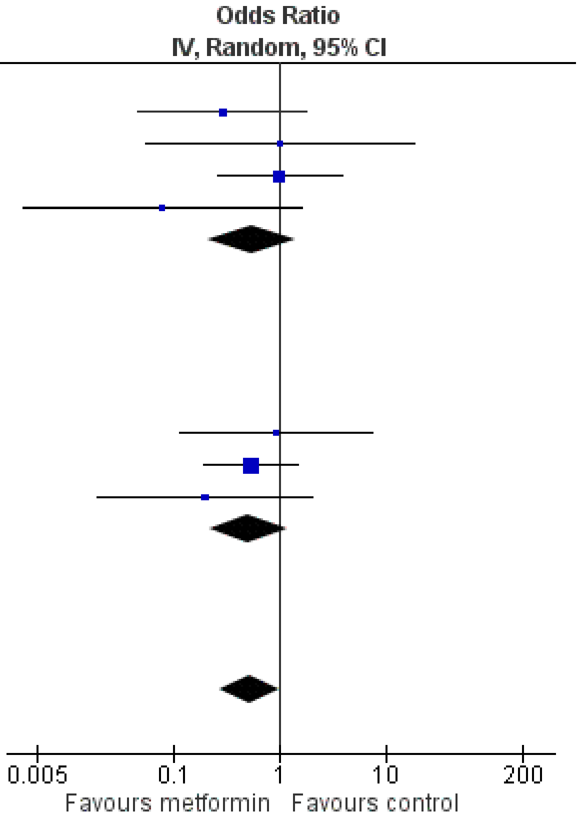

Figure E: Forest plot of the effect of metformin on mortality, stratified by comorbidity (yes/no). Data on mortality in metformin-treated groups and control groups was extracted from primary studies and expressed as odds ratios (blue squares). The pooled effect estimates (diamonds) show no effect of metformin on mortality in healthy or comorbid animals. There was no difference between the subgroups. SD = standard deviation, Total = sample size, CI = confidence interval, IV = inverse variance, HFD = high fat diet, ND = normal diet, DM = diabetes mellitus, permI = permanent ischemia, 28xM = 28 doses of metformin in total.

Study or Subgroup	Metformin Events	Metformin Total	Control Events	Control Total	Weight	Odds Ratio IV, Random, 95% CI
**1.12.3 Metformin pre I**						
Apaijai2014_HFD	2	16	5	15	12.3%	0.29 [0.05, 1.78]
Apaijai2014_ND	2	16	2	15	9.4%	0.93 [0.11, 7.59]
NoyanAshraf2009_DMHFD	7	18	7	18	23.0%	1.00 [0.26, 3.82]
Sauve2010_DMHFD	0	16	4	15	4.5%	0.08 [0.00, 1.58]
**Subtotal (95% CI)**		**66**		**63**	**49.2%**	**0.56 [0.22, 1.44]**
Total events	11		18			
Heterogeneity: Tau² = 0.04; Chi² = 3.11, df = 3 (P = 0.37); I² = 4%						
Test for overall effect: Z = 1.20 (P = 0.23)						
**1.12.4 Metformin during I / at R**						
Elmadhun2013	1	9	1	9	4.8%	1.00 [0.05, 18.91]
Gundewar2009_permI_28xM	15	27	26	37	38.4%	0.53 [0.19, 1.49]
Wang2011	1	15	4	15	7.6%	0.20 [0.02, 2.02]
**Subtotal (95% CI)**		**51**		**61**	**50.8%**	**0.48 [0.20, 1.19]**
Total events	17		31			
Heterogeneity: Tau² = 0.00; Chi² = 0.84, df = 2 (P = 0.66); I² = 0%						
Test for overall effect: Z = 1.58 (P = 0.11)						
**Total (95% CI)**		**117**		**124**	**100.0%**	**0.52 [0.28, 1.00]**
Total events	28		49			
Heterogeneity: Tau² = 0.00; Chi² = 4.01, df = 6 (P = 0.68); I² = 0%						
Test for overall effect: Z = 1.97 (P = 0.05)						
Test for subgroup differences: Chi² = 0.05, df = 1 (P = 0.82), I² = 0%						

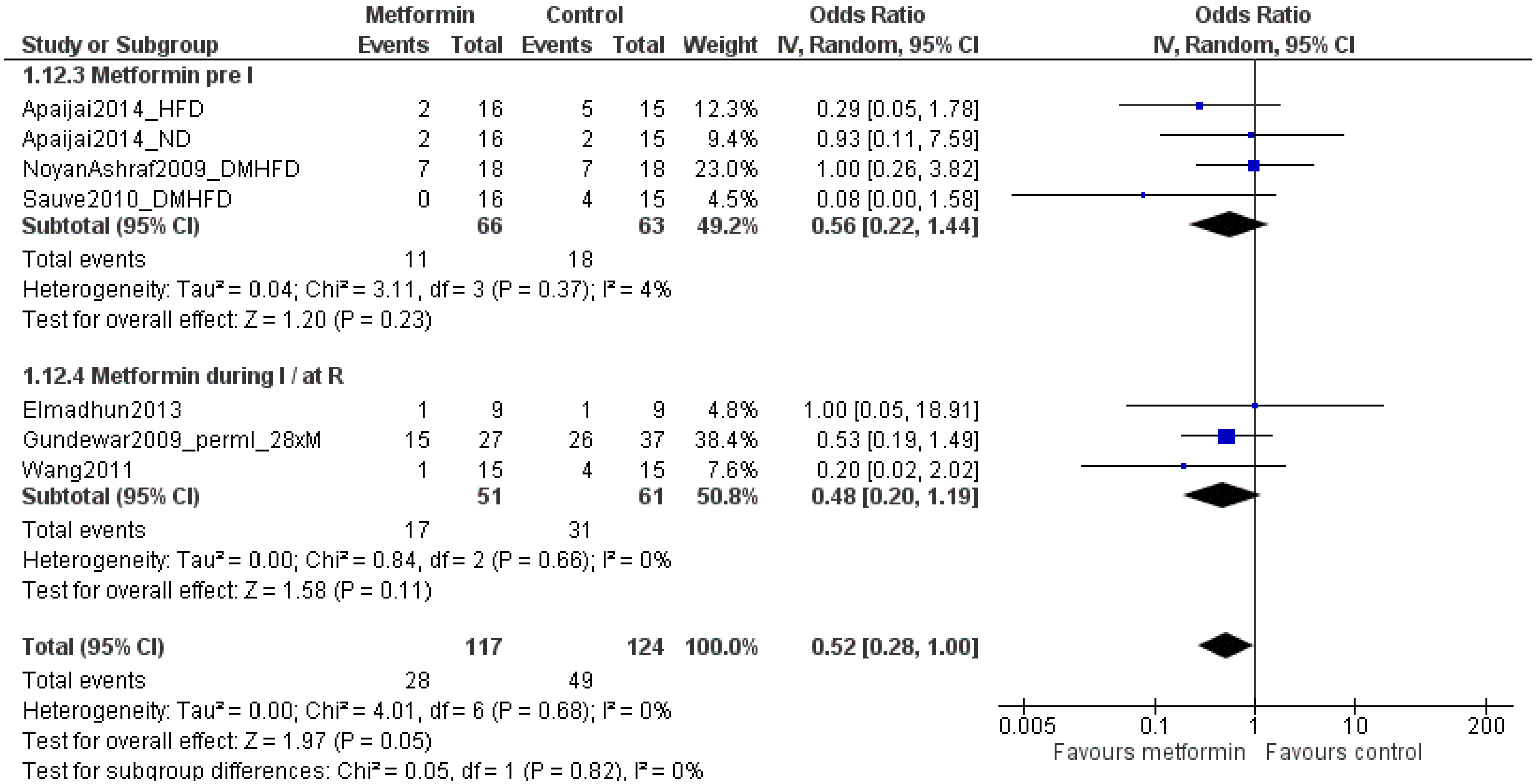

Figure F: Forest plot of the effect of metformin on mortality, stratified by timing of treatment. Data on mortality in metformin-treated groups and control groups was extracted from primary studies and expressed as odds ratios (blue squares). The pooled effect estimates (diamonds) show no effect of metformin when given before ischemia (pre I), nor when given during ischemia or at reperfusion (during I / at R). There was no difference between the subgroups. Total = sample size, CI = confidence interval, IV = inverse variance, HFD = high fat diet, ND = normal diet, DM = diabetes mellitus, permI = permanent ischemia, 28xM = 28 doses of metformin in total.
